# ﻿Gap analysis of knowledge about the microstructure of *Impatiens* Riv. ex L. (Balsaminaceae) seeds using SEM techniques

**DOI:** 10.3897/phytokeys.262.152205

**Published:** 2025-09-02

**Authors:** Agnieszka Rewicz, René Monzalvo, Kayla Grey, Katarzyna Sanek, Maja Mackiewicz, Monika Myśliwy

**Affiliations:** 1 Department of Geobotany and Plant Ecology, University of Lodz, Banacha 12/16, 90-237, Łódź, Poland; 2 School of Biological Sciences, Southern Illinois University, 1125 Lincoln Drive, Carbondale, IL 62901-6501, USA; 3 School of Forestry and Horticulture, School of Agricultural Sciences. College of Agriculture, Life and Physical Sciences, Southern Illinois University, 1205 Lincoln Dr, Carbondale, IL 62901-6501, USA; 4 Center for Forensic Science University of Warsaw, Żwirki i Wigury 101, 02-089 Warsaw, Poland; 5 Department of Environmental Ecology, Institute of Marine and Environmental Sciences, University of Szczecin, Adama Mickiewicza 16, 70-383 Szczecin, Poland

**Keywords:** Micromorphological analysis, plant taxonomy, seed coat, systematic studies, taxonomic classification

## Abstract

We conducted a literature review on the taxonomic importance of seed micromorphology in the genus *Impatiens* Riv. ex L. (Balsaminaceae), focusing on studies using scanning electron microscopy (SEM). Of 47 sources, SEM descriptions were available for more than 180 species, revealing significant gaps in micromorphological data within the genus. Our work provides a consolidated resource on *Impatiens* taxonomy and highlights the need for further SEM studies to improve species identification and phylogenetic analysis.

## ﻿Introduction

Owing to differences in the understanding of taxonomic features across different groups of plants, we can more accurately assess the usefulness and level of conservatism of specific taxonomic traits within plant groups. Undoubtedly, among the most important taxonomic features are those related to flowers (e.g. in Ranunculaceae, Asteraceae families) (Hoots 1991; Karanović et al. 2015; Zhai et al. 2019), and examples of their use can be found at all levels of the taxonomic hierarchy. Conversely, seed and fruit characters are less prevalent and indeed possess lesser taxonomic significance. However, there are botanical families where carpological research is the most useful taxonomic feature (e.g. in Caryophyllaceae or Brassicaceae families) (Abdel-Maksoud and Fawzi 2016; Ullah et al. 2019; Gabr 2018; Hashem AL-Blesh et al. 2021). An example is Caryophyllaceae, whose features such as tubercle shape, size, and distribution provide key diagnostic characters (Martín-Gómez et al. 2024a; 2024b). Unusual forms and arrangements include echinate, rugose, mammillate, papillose, and smooth seed tubercles, as well as epidermal cell wall patterns such as digitate, serrate, sinuous, and rectangular (Hubinský et al. 2024). But a crucial aspect lies in the extent of research into the micromorphology of seeds and fruits. Historically, descriptions of seeds and fruits were based on observations through light microscopes or binoculars, often failing to highlight interesting features of their structure, hence their role remained underappreciated by botanists (Hooker 1904–1906, 1909; Tardieu-Blot 1945–1946; Perrier de la Bathie 1948). Another challenge in carpological research was the limited availability of material. Botanists, typically emphasizing species descriptions, often focused on flowering specimens, and seeds were not always available on these specimens. It should also be remembered that only mature seeds or fruits can be the basis for correct taxonomic identification.

Thanks to progress in microscopy techniques, particularly the utilization of SEM (scanning electron microscope), research on seed and fruit cover has become more common, offering new perspectives and approaches to establishing boundaries between organisms (Brisson and Peterson 1976; Godfray and Knapp 2004). The first carpological research using this technology took place in the mid-1960s, but the real development of the use of SEM in botany, including carpology, occurred only in the 1990s (Barthlott 1981, 1984). This, in turn, resulted in the discovery of unusual forms, shapes and arrangements in known seed structures (e. g., net, reticulated and papillated-like patterns in the primary and secondary sculpture of cell walls) (Barthlott 1981) drawing attention to the fact that these features could be of significant importance in plant taxonomy (Heywood 1969; Stace 1992). Currently, an integrated approach to taxonomy can contribute to solving difficult or complicated cases (Ruchisansakun et al. 2015; Yu et al. 2015; Rahelivololona et al. 2018). Literature data indicate that seed and fruit characteristics serve as valuable tools at various taxonomic levels including families, e.g. Brassicaceae, Apiaceae, Rosaceae, and Caryophyllaceae (Crow 1979; Abdel-Maksoud and Fawzi 2016), genera, e.g. *Silene* (Fawzi et al. 2010; Atazadeh et al. 2017), *Dianthus*, *Spergula*, and *Spergularia* (Gvinianidze and Fedotova 1991), and even subspecies such as in *Montia fontana*, where they are solely distinguished based on seed morphology (Heywood 1974).

However, to ascertain the significance of seeds and fruits as a valuable tool in the taxonomy of a given group, thorough knowledge and description of these features are imperative. Therefore, the contemporary challenge in carpology lies not only in filling the knowledge gaps concerning various plant groups, but also in specifying how seed images are used in taxonomy. Both light microscopy and scanning electron microscopy (SEM) play an essential role in this context. Standard morphometric analyses typically include measurements of seed length, width, surface area, perimeter, and length-to-width ratio (aspect ratio), providing a fundamental quantitative basis for comparing taxa (Zhai et al. 2019; Martín-Gómez et al. 2024a). Moreover, seed shape analysis – increasingly performed using geometric morphometrics and elliptic Fourier analysis – opens new avenues for taxonomic and evolutionary studies, enabling objective comparisons across large datasets (Martín-Gómez et al. 2023). In addition to these parameters, SEM images allow for the detailed observation of seed coat cell arrangement and shape, as well as the morphology of periclinal and anticlinal cell walls, which serve as valuable diagnostic characters in species identification and classification (Rewicz et al. 2022c). In this work, our focus is on the genus *Impatiens*, which has been extensively studied in terms of systematics, ecology, and morphology. *Impatiens* is distinguished by its enormous species richness and extremely variable flowers, which makes it one of the most difficult groups to classify (Song et al. 2005; Cai et al. 2007; Janssens et al. 2010; Bhaskar 2012; Gogoi et al. 2018). New species are constantly being identified in various regions of the world (Fujimoto et al. 2024; Paing et al. 2024; Song et al. 2024), and research on them emphasizes the importance of seed micromorphology as an important feature in the process of species identification (Shimizu 1979; Lu and Chen 1991). Moreover, the ultrastructure of seeds can provide information about the phylogenetic relationships between them (Song et al. 2003, 2005; Yuan et al. 2004). It has been demonstrated that seed shape and seed coat micromorphology are effective tools for resolving taxonomic issues, including within the Balsaminaceae family, to which the genus *Impatiens* belongs (cf. Utami and Shimizu 2005; Chen et al. 2007; Cai et al. 2013; Zhang et al. 2016). Detailed morphometric analyses commonly involve measurements of seed length and width. In addition, scanning electron microscopy is employed to examine the ultrastructure of the testa, with particular attention to diagnostic characters such as the shape and arrangement of epidermal cells, the nature of anticlinal and periclinal walls, and specific surface ornamentation patterns. According to Zhang et al. (2016), features like reticulate or striate ornamentation, variations in cell wall elevation, and granule presence on the testa surface can be critical in differentiating species and understanding their phylogenetic relationships. Despite this, there is still much to be done, as the micromorphology of the seed coat has been studied using SEM in less than one fifth of *Impatiens* species (Maciejewska-Rutkowska and Janczak 2016). Literature data have shown that descriptions of *Impatiens* spp. seeds are typically brief, accompanied by microscopic observations, and sometimes supplemented with measurements, photographs or diagrams (Suppl. material 1). However, the absence of crucial details, such as measurements or SEM images, poses challenges for comprehensive analysis (Abid et al. 2011). In this study, we analyzed the gaps in available literature concerning the micromorphology of seeds in the genus *Impatiens* using SEM. The aim of our work was to develop the current state of knowledge on this issue, thus providing researchers with a comprehensive literature review on the ultrastructure of seeds.

## ﻿Material and methods

A complete list of species of the genus *Impatiens* that have studies on seed carpology was prepared on the basis of available literature data, including information published in scientific articles and data from “gray literature”, such as local monographs and technical reports (Table 1). Our primary inclusion criteria comprised studies exclusively involving SEM analyses that incorporate SEM images. Subsequently, we gathered data on the missing species (species with no SEM images) considering the following variables: a) morphological description of the seeds, imagery including microscope and binocular photos, drawings and graphics and b) the geographical origin of the analyzed material (country) (Supp. material 1).

**Table 1.** List of species of the genus *Impatiens* with SEM images of seeds.

Number	Species name	Source	Remarks
1	*Impatiens acaulis* Arn.	Utami and Shimizu 2005	accepted by WFO (placed as the accepted name of a taxon)
2	*Impatiens aconitoides* Y.M.Shui & W.H.Chen	Shui et al. 2011	accepted by WFO (placed as the accepted name of a taxon)
3	*Impatiens alboflava* Miq.	Utami and Shimizu 2005	accepted by WFO (placed as the accepted name of a taxon)
4	*Impatiens alpicola* Y.L.Chen & Y.Q.Lu	Lu and Chen 1991	accepted by WFO (placed as the accepted name of a taxon)
5	*Impatiens anjawensis* Borah, Kandwal, Chhetri & Gogoi	Rewicz et al. 2020a	accepted by WFO (placed as the accepted name of a taxon)
6	*Impatiens apalophylla* Hook.f.	Yu et al. 2015	accepted by WFO (placed as the accepted name of a taxon)
7	*Impatiens apsotis* Hook.f.	Song et al. 2005	accepted by WFO (placed as the accepted name of a taxon)
8	*Impatiens aquatilis* Hook.f.	Song et al. 2005	accepted by WFO (placed as the accepted name of a taxon)
9	*Impatiens aquatilis* Hook.f.	Zhang et al. 2016	accepted by WFO (placed as the accepted name of a taxon)
10	*Impatiens aquatilis* Hook.f.	Xia et al. 2019	accepted by WFO (placed as the accepted name of a taxon)
11	*Impatiens arguta* Hook.f. & Thomson	Cai et al. 2013	accepted by WFO (placed as the accepted name of a taxon)
12	*Impatiens arguta* Hook.f. & Thomson	Song et al. 2005	accepted by WFO (placed as the accepted name of a taxon)
13	*Impatiens arriensii* (*alliensii*) (Zoll.) T. Shimizu	Shimizu 1987	accepted by WFO (placed as the accepted name of a taxon)
14	*Impatiens arriensii* (Zoll.) T.Shimizu	Utami and Shimizu 1995	accepted by WFO (placed as the accepted name of a taxon)
15	*Impatiens aureliana* Hook.f.	Cai et al. 2013	synonym of *Impatiens violiflora* Hook.f.
16	*Impatiens aureliana* Hook.f.	Song et al. 2005	synonym of *Impatiens violiflora* Hook.f.
17	*Impatiens badrinathii* Pusalkar & D.K.Singh	Pusalkar and Singh 2010	synonym of *Impatiens chungtienensis* Y.L.Chen
18	*Impatiens bahanensis* Hand.- Mazz.	Zhang et al. 2016	accepted by WFO (placed as the accepted name of a taxon)
19	*Impatiens bahanensis* Hand.-Mazz.	Song et al. 2005	accepted by WFO (placed as the accepted name of a taxon)
20	? *Impatiens baishaensis* B.Ding & H. P. Deng	Ding et al. 2017	not found on WFO
21	*Impatiens balfourii* Hook. f.	Najberek et al. 2020	accepted by WFO (placed as the accepted name of a taxon)
22	*Impatiens balfourii* Hook. f.	Song et al. 2005	accepted by WFO (placed as the accepted name of a taxon)
23	*Impatiens balsamina* L.	Song et al. 2005	accepted by WFO (placed as the accepted name of a taxon)
24	*Impatiens balsamina* L.	Utami and Shimizu 2005	accepted by WFO (placed as the accepted name of a taxon)
25	*Impatiens balsamina* L.	Chen et al. 2007	accepted by WFO (placed as the accepted name of a taxon)
26	*Impatiens barbata* Comber	Yu et al. 2015	accepted by WFO (placed as the accepted name of a taxon)
27	*Impatiens begoniifolia* S.Akiyama & H.Ohba	Song et al. 2005	accepted by WFO (placed as the accepted name of a taxon)
28	*Impatiens benthamii* Steenis	Utami and Shimizu 2005	accepted by WFO (placed as the accepted name of a taxon)
29	*Impatiens bicolor* subsp. *Bicolor*	Abid et al. 2011	synonym of *Impatiens bicolor* Royle
30	Impatiens bicolor Royle subsp. pseudo-bicolor (Grey-Wilson) Y.J.Nasir	Abid et al. 2011	synonym of *Impatiens bicolor* Royle
31	*Impatiens bicornis* L.Joseph & Bhaskar	Bhaskar 2012	accepted by WFO (placed as the accepted name of a taxon)
32	*Impatiens blepharosephala* E.Pritz.	Cai et al. 2013	accepted by WFO (placed as the accepted name of a taxon)
33	*Impatiens blinii* H.Lév.	Song et al. 2022	accepted by WFO (placed as the accepted name of a taxon)
34	*Impatiens bodinieri* Hook.f.	Song et al. 2022	accepted by WFO (placed as the accepted name of a taxon)
35	*Impatiens brachycentra* Kar. & Kir.	Zhang et al. 2016	accepted by WFO (placed as the accepted name of a taxon)
36	? *Impatiens brachycentra* Kar. & Kir. *var. jacquemontii* (Hook.f.)	Abid et al. 2011	not found on WFO
37	*Impatiens burtonii* Hook.f.	Song et al. 2022	accepted by WFO (placed as the accepted name of a taxon)
38	*Impatiens capensis* Meerb.	Rewicz et al. 2020b	accepted by WFO (placed as the accepted name of a taxon)
39	*Impatiens chekiangensis* Y.L.Chen	Song et al. 2022	accepted by WFO (placed as the accepted name of a taxon)
40	*Impatiens chimiliensis* Comber	Cai et al. 2013	accepted by WFO (placed as the accepted name of a taxon)
41	*Impatiens chinensis* L.	Chen et al. 2007	accepted by WFO (placed as the accepted name of a taxon)
42	*Impatiens chinensis* L.	Song et al. 2005	accepted by WFO (placed as the accepted name of a taxon)
43	*Impatiens chinensis* L.	Utami and Shimizu 2005	accepted by WFO (placed as the accepted name of a taxon)
44	*Impatiens chloroxantha* Y.L.Chen	Wang et al. 2020	accepted by WFO (placed as the accepted name of a taxon)
45	*Impatiens chungtienensis* Y.L.Chen	Song et al. 2005	accepted by WFO (placed as the accepted name of a taxon)
46	*Impatiens clavigera* Hook.f.	Song et al. 2022	accepted by WFO (placed as the accepted name of a taxon)
47	Impatiens clavigera var. auriculata S.H.Huang	Lei et al. 2015	synonym of *Impatiens apalophylla* Hook.f
48	*Impatiens commelinoides* Hand.-Mazz.	Song et al. 2022	accepted by WFO (placed as the accepted name of a taxon)
49	*Impatiens conchibracteata* Y.L.Chen & Y.Q.Lu	Lu and Chen 1991	accepted by WFO (placed as the accepted name of a taxon)
50	*Impatiens cordata* Wight	Utami and Shimizu 2005	accepted by WFO (placed as the accepted name of a taxon)
51	*Impatiens cornutisepala* S.X.Yu, Y.L.Chen & H.N.Qin	Yu et al. 2009	accepted by WFO (placed as the accepted name of a taxon)
52	*Impatiens cyanantha* Hook.f.	Song et al. 2022	accepted by WFO (placed as the accepted name of a taxon)
53	*Impatiens cyathiflora* Hook.f.	Song et al. 2022	accepted by WFO (placed as the accepted name of a taxon)
54	*Impatiens cymbifera* Hook.f.	Zhang et al. 2016	accepted by WFO (placed as the accepted name of a taxon)
55	*Impatiens damingensis* S.X.Yu, Chang Y. Xia & H. P.Deng	Xia et al. 2019	accepted by WFO (placed as the accepted name of a taxon)
56	*Impatiens davidii* Franch.	Chen et al. 2007	accepted by WFO (placed as the accepted name of a taxon)
57	*Impatiens davidii* Franch.	Song et al. 2005	accepted by WFO (placed as the accepted name of a taxon)
58	*Impatiens decipiens* Hook.f.	Rewicz et al. 2020a	accepted by WFO (placed as the accepted name of a taxon)
59	*Impatiens delavayi* Franch.	Yu et al. 2022	accepted by WFO (placed as the accepted name of a taxon)
60	*Impatiens delavayi* Franch.	Song et al. 2005	accepted by WFO (placed as the accepted name of a taxon)
61	*Impatiens devendrae* Pusalkar	Pusalkar and Singh 2010	accepted by WFO (placed as the accepted name of a taxon)
62	*Impatiens dicentra* Franch. ex Hook.f.	Song et al. 2021c	accepted by WFO (placed as the accepted name of a taxon)
63	*Impatiens dicentra* Franch. ex Hook.f.	Chen et al. 2007	accepted by WFO (placed as the accepted name of a taxon)
64	*Impatiens dolichoceras* E.Pritz. ex Diels	Song et al. 2005	accepted by WFO (placed as the accepted name of a taxon)
65	*Impatiens drepanophora* Hook.f.	Song et al. 2022	accepted by WFO (placed as the accepted name of a taxon)
66	*Impatiens drepanophora* Hook.f.	Rewicz et al. 2020a	accepted by WFO (placed as the accepted name of a taxon)
67	*Impatiens edgeworthii* Hook.f.	Abid et al. 2011	accepted by WFO (placed as the accepted name of a taxon)
68	*Impatiens elwiraurzu-lae* Eb. Fisch., Abrah., Holstein & S.B. Janssens	Fischer et al. 2021	accepted by WFO (placed as the accepted name of a taxon)
69	*Impatiens faberi* Hook.f.	Lu and Chen 1991	accepted by WFO (placed as the accepted name of a taxon)
70	*Impatiens falcifera* Hook.f.	Zhang et al. 2016	accepted by WFO (placed as the accepted name of a taxon)
71	*Impatiens fanjingshanica* Y.L.Chen	Cai et al. 2013	accepted by WFO (placed as the accepted name of a taxon)
72	*Impatiens fenghwaiana* Y.L.Chen	Song et al. 2005	accepted by WFO (placed as the accepted name of a taxon)
73	*Impatiens flemingii* Hook.f.	Abid et al. 2011	accepted by WFO (placed as the accepted name of a taxon)
74	*Impatiens forrestii* Hook.f. & W.W. Sm.	Song et al. 2005	accepted by WFO (placed as the accepted name of a taxon)
75	*Impatiens fragicolor* C.Marquand & Airy Shaw	Cai et al. 2013	accepted by WFO (placed as the accepted name of a taxon)
76	*Impatiens fragicolor* C.Marquand & Airy Shaw	Zhang et al. 2016	accepted by WFO (placed as the accepted name of a taxon)
77	*Impatiens fugongensis* K.M.Liu & Y.Y.Cong	Cai et al. 2013	accepted by WFO (placed as the accepted name of a taxon)
78	*Impatiens fugongensis* K.M.Liu & Y.Y.Cong	Cong et al. 2008	accepted by WFO (placed as the accepted name of a taxon)
79	*Impatiens galactica* Eb.Fisch., Raheliv. & Abrah.	Fischer et al. 2017	accepted by WFO (placed as the accepted name of a taxon)
80	*Impatiens gardneriana* Wight.	Utami and Shimizu 2005	accepted by WFO (placed as the accepted name of a taxon)
81	*Impatiens garrettii* Craib	Utami and Shimizu 2005	synonym of *Impatiens porrecta* Wall. ex Hook.f. & Thomson
82	*Impatiens gesneroidea* Gilg	Fischer et al. 2021	accepted by WFO (placed as the accepted name of a taxon)
83	*Impatiens glandulifera* Royle	Najberek et al. 2020	accepted by WFO (placed as the accepted name of a taxon)
84	*Impatiens glandulifera* Royle	Abid et al. 2011	accepted by WFO (placed as the accepted name of a taxon)
85	*Impatiens glandulifera* Royle	Maciejewska-Rutkowska and Janczak 2016	accepted by WFO (placed as the accepted name of a taxon)
86	*Impatiens gongshanensis* Y.L.Chen	Song et al. 2005	accepted by WFO (placed as the accepted name of a taxon)
87	*Impatiens goughii* Wight	Utami and Shimizu 2005	accepted by WFO (placed as the accepted name of a taxon)
88	*Impatiens guizhouensis* Y.L.Chen	Song et al. 2022	accepted by WFO (placed as the accepted name of a taxon)
89	*Impatiens hainanensis* Y.L.Chen	Bi et al. 2010	accepted by WFO (placed as the accepted name of a taxon)
90	*Impatiens harae* H.Ohba & S. Akiyama	Zhang et al. 2016	accepted by WFO (placed as the accepted name of a taxon)
91	*Impatiens henslowiana* Arn.	Utami and Shimizu 2005	accepted by WFO (placed as the accepted name of a taxon)
92	*Impatiens hirta* L.Joseph & Bhaskar	Bhaskar 2012	accepted by WFO
93	*Impatiens holocentra* Hand.-Mazz.	Cai et al. 2013	accepted by WFO (placed as the accepted name of a taxon)
94	Impatiens huangyanensis X.F.Jin & B.Y.Ding subsp. attenuata X.F.Jin & Z.H.Chen	Song et al. 2022	accepted by WFO (placed as the accepted name of an infraspecific taxon of the species *Impatiens huangyanensis* X.F.Jin & B.Y.Ding)
95	Impatiens huangyanensis X.F.Jin & B.Y.Ding subsp. attenuata X.F.Jin & Z.H.Chen	Jin et al. 2008	accepted by WFO (placed as the accepted name of an infraspecific taxon of the species *Impatiens huangyanensis* X.F.Jin & B.Y.Ding)
96	Impatiens huangyanensis X.F.Jin & B.Y.Ding subsp. huangyanensis	Jin et al. 2008	accepted by WFO (placed as the accepted name of an infraspecific taxon of the species *Impatiens huangyanensis* X.F.Jin & B.Y.Ding)
97	*Impatiens hunanensis* Y.L.Chen	Song et al. 2022	accepted by WFO (placed as the accepted name of a taxon)
98	*Impatiens imbecilla* Hook.f.	Lu and Chen 1991	accepted by WFO (placed as the accepted name of a taxon)
99	*Impatiens inﬁrma* Hook.f.	Song et al. 2005	accepted by WFO (placed as the accepted name of a taxon)
100	*Impatiens jenjittikuliae* Ruchis. & Suksathan	Ruchisansakun and Suksathan 2019	accepted by WFO (placed as the accepted name of a taxon)
101	*Impatiens jinggangensis* Y.L.Chen	Cai et al. 2013	accepted by WFO (placed as the accepted name of a taxon)
102	*Impatiens jinpingensis* Y.M.Shui & G.F.Li	Song et al. 2022	accepted by WFO (placed as the accepted name of a taxon)
103	*Impatiens jurpia* Buch.-Ham. ex Hook.f. & Thomson	Rewicz et al. 2020a	accepted by WFO (placed as the accepted name of a taxon)
104	*Impatiens kivuensis* Eb.Fisch., Abrah., Holstein & S.B.Janssens	Fischer et al. 2021	accepted by WFO (placed as the accepted name of a taxon)
105	*Impatiens larsenii* T.Shimizu	Utami and Shimizu 2005	accepted by WFO (placed as the accepted name of a taxon)
106	*Impatiens lateristachys* Y.L.Chen & Y.Q.Lu	Lu and Chen 1991	accepted by WFO (placed as the accepted name of a taxon)
107	*Impatiens latipetala* S.H.Huang	Song et al. 2022	accepted by WFO (placed as the accepted name of a taxon)
108	*Impatiens laxiflora* Edgew.	Zhang et al. 2016	accepted by WFO (placed as the accepted name of a taxon)
109	*Impatiens leggei* Pusalkar & D.K.Singh	Pusalkar and Singh 2010	synonym of *Impatiens laxiflora* Edgew.
110	Impatiens lemannii Hook.f. & Thomson subsp. kurramensis Grey-Wilson	Abid et al. 2011	accepted by WFO (placed as the accepted name of an infraspecific taxon of the species *Impatiens lemannii* Hook.f. & Thomson)
111	Impatiens lemannii Hook.f. & Thomson subsp. lemannii	Abid et al. 2011	accepted by WFO (placed as the accepted name of an infraspecific taxon of the species *Impatiens lemannii* Hook.f. & Thomson)
112	*Impatiens lemeei* H.Lév.	Chen et al. 2007	accepted by WFO (placed as the accepted name of a taxon)
113	*Impatiens lepida* Hook.f.	Song et al. 2022	accepted by WFO (placed as the accepted name of a taxon)
114	*Impatiens leptocaulon* Hook.f	Song et al. 2022	accepted by WFO (placed as the accepted name of a taxon)
115	*Impatiens liboensis* K.M.Liu & R.P.Kuang	Kuang et al. 2014	accepted by WFO (placed as the accepted name of a taxon)
116	*Impatiens linearisepala* S.Akiyama, H.Ohba & S.K.Wu	Song et al. 2022	accepted by WFO (placed as the accepted name of a taxon)
117	*Impatiens lobulifera* S.X.Yu, Y.L.Chen & H.N.Qin	Yu et al. 2009	accepted by WFO (placed as the accepted name of a taxon)
118	*Impatiens longialata* E.Pritz.	Song et al. 2022	accepted by WFO (placed as the accepted name of a taxon)
119	*Impatiens longicornuta* Y.L.Chen	Cai et al. 2013	accepted by WFO (placed as the accepted name of a taxon)
120	*Impatiens longipes* Hook.f. & Thomson	Zhang et al. 2016	accepted by WFO (placed as the accepted name of a taxon)
121	*Impatiens longirostris* S.H.Huang	Cai et al. 2013	accepted by WFO (placed as the accepted name of a taxon)
122	^*Impatiens longshanensis* Y.Y.Cong & Y.X.Song	Song et al. 2021c	unplaced by WFO (this name is currently unchecked and awaiting taxonomic scrutiny), found on IPNI
123	*Impatiens ludewigii* Eb.Fisch., Abrah., Holstein & S.B.Janssens	Fischer et al. 2021	accepted by WFO (placed as the accepted name of a taxon)
124	*Impatiens lutzmanniae* Eb.Fisch., Abrah., Holstein & S.B.Janssens	Fischer et al. 2021	accepted by WFO (placed as the accepted name of a taxon)
125	*Impatiens macrovexilla var. macrovexilla*	Yu 2007	accepted by WFO (placed as the accepted name of an infraspecific taxon of the species *Impatiens macrovexilla* Y.L.Chen)
126	*Impatiens macrovexilla Y.L.Chen var. yaoshanensis* S.X.Yu, Y.L.Chen & H.N.Qin	Yu 2007	accepted by WFO (placed as the accepted name of an infraspecific taxon of the species *Impatiens macrovexilla* Y.L.Chen)
127	*Impatiens maculifera* S.X.Yu & Chang Y.Xia	Song et al. 2022	accepted by WFO (placed as the accepted name of a taxon)
128	*Impatiens maculifera* S.X.Yu & Chang Y.Xia	Xia et al. 2019	accepted by WFO (placed as the accepted name of a taxon)
129	*Impatiens margaritifera* Hook.f.	Zhang et al. 2016	accepted by WFO (placed as the accepted name of a taxon)
130	*Impatiens menghuochengensis* Q.Luo	Luo et al. 2014	accepted by WFO (placed as the accepted name of a taxon)
131	*Impatiens mengtszeana* Hook.f.	Song et al. 2005	synonym of *Impatiens pulchra* Hook.f. & Thomson.; accepted name in the genus *Impatiens*
132	*Impatiens meruensis* Gilg	Song et al. 2022	accepted by WFO (placed as the accepted name of a taxon)
133	*Impatiens meyana* Hook.f.	Song et al. 2022	accepted by WFO (placed as the accepted name of a taxon)
134	*Impatiens monticola* Hook.f.	Cai et al. 2013	synonym of *Impatiens pulchra* Hook.f. & Thomson.; accepted name in the genus *Impatiens*
135	*Impatiens monticola* Hook.f.	Song et al. 2022	synonym of *Impatiens pulchra* Hook.f. & Thomson.; accepted name in the genus *Impatiens*
136	*Impatiens monticola* Hook.f.	Lu and Chen 1991	synonym of *Impatiens pulchra* Hook.f. & Thomson.; accepted name in the genus *Impatiens*
137	*Impatiens monticola* Hook.f.	Cong et al. 2008	synonym of *Impatiens pulchra* Hook.f. & Thomson.; accepted name in the genus *Impatiens*
138	*Impatiens morsei* Hook.f.	Yu et al. 2015	accepted by WFO (placed as the accepted name of a taxon)
139	*Impatiens morsei* Hook.f.	Yu et al. 2009	accepted by WFO (placed as the accepted name of a taxon)
140	^*Impatiens muscicola* Craib	Utami and Shimizu 2005	unplaced by WFO (this name is currently unchecked and awaiting taxonomic scrutiny), found on IPNI
141	*Impatiens nagorum* Gogoi, Moaakum & S.Dey	Moaakum et al. 2017	accepted by WFO (placed as the accepted name of a taxon)
142	*Impatiens namkatensis* T.Shimizu	Utami and Shimizu 2005	accepted by WFO (placed as the accepted name of a taxon)
143	*Impatiens napoensis* Y.L.Chen	Cai et al. 2013	accepted by WFO (placed as the accepted name of a taxon)
144	*Impatiens nasuta* H.Perrier	Song et al. 2022	synonym of *Impatiens kanchigandhiana* Rasingam, Karthig. & Gogoi.; accepted name in the genus *Impatiens*
145	*Impatiens noli-tangere* L.	Song et al. 2022	accepted by WFO (placed as the accepted name of a taxon)
146	*Impatiens noli-tangere* L.	Song et al. 2005	accepted by WFO (placed as the accepted name of a taxon)
147	*Impatiens noli-tangere* L.	Utami and Shimizu 2005	accepted by WFO (placed as the accepted name of a taxon)
148	*Impatiens noli-tangere* L.	Chen et al. 2007	accepted by WFO (placed as the accepted name of a taxon)
149	*Impatiens noli-tangere* L.	Jin et al. 2008	accepted by WFO (placed as the accepted name of a taxon)
150	*Impatiens nyimana* C.Marquand & Airy Shaw	Rewicz et al. 2020a	accepted by WFO (placed as the accepted name of a taxon)
151	*Impatiens nyimana* C.Marquand & Airy Shaw	Zhang et al. 2016	accepted by WFO (placed as the accepted name of a taxon)
152	*Impatiens obesa* Hook.f.	Shimizu 1987	accepted by WFO (placed as the accepted name of a taxon)
153	*Impatiens obesa* Hook.f.	Song et al. 2022	accepted by WFO (placed as the accepted name of a taxon)
154	*Impatiens obesa* Hook.f.	Bi et al. 2010	accepted by WFO (placed as the accepted name of a taxon)
155	*Impatiens oblongipetala* K.M.Liu & Y.Y.Cong	Cai et al. 2013	accepted by WFO (placed as the accepted name of a taxon)
156	*Impatiens oblongipetala* K.M.Liu & Y.Y.Cong	Cong et al. 2010	accepted by WFO (placed as the accepted name of a taxon)
157	*Impatiens omeiana* Hook.f.	Song et al. 2022	accepted by WFO (placed as the accepted name of a taxon)
158	*Impatiens omissa* Hook.f.	Utami and Shimizu 2005	accepted by WFO (placed as the accepted name of a taxon)
159	*Impatiens oxyanthera* Hook.f.	Lu and Chen 1991	accepted by WFO (placed as the accepted name of a taxon)
160	*Impatiens parviﬂora* DC.	Song et al. 2005	accepted by WFO (placed as the accepted name of a taxon)
161	^*Impatiens patula* Craib	Utami and Shimizu 2005	unplaced by WFO (this name is currently unchecked and awaiting taxonomic scrutiny), found on IPNI
162	*Impatiens pianmaensis* S.H.Huang	Song et al. 2022	accepted by WFO (placed as the accepted name of a taxon)
163	*Impatiens pinetorum* Hook.f. ex W.W.Sm.	Cai et al. 2013	accepted by WFO (placed as the accepted name of a taxon)
164	*Impatiens pingxiangensis* H.Y.Bi & S.X.Yu	Bi et al. 2010	accepted by WFO (placed as the accepted name of a taxon)
165	*Impatiens piufanensis* Hook.f.	Chen et al. 2007	accepted by WFO (placed as the accepted name of a taxon)
166	*Impatiens platychlaena* Hook.f.	Lu and Chen 1991	accepted by WFO (placed as the accepted name of a taxon)
167	*Impatiens platysepala* Y.L.Chen	Yu et al. 2015	accepted by WFO (placed as the accepted name of a taxon)
168	*Impatiens platysepala* Y.L.Chen	Wang et al. 2020	accepted by WFO (placed as the accepted name of a taxon)
169	*Impatiens poculifer* Hook.f.	Song et al. 2005	accepted by WFO (placed as the accepted name of a taxon)
170	*Impatiens polyneura* K.M.Liu	Song et al. 2022	accepted by WFO (placed as the accepted name of a taxon)
171	*Impatiens polyneura* K.M.Liu	Chen et al. 2007	accepted by WFO (placed as the accepted name of a taxon)
172	*Impatiens principis* Hook.f.	Song et al. 2022	accepted by WFO (placed as the accepted name of a taxon)
173	*Impatiens principis* Hook.f.	Zhang et al. 2016	accepted by WFO (placed as the accepted name of a taxon)
174	*Impatiens pritzelii* Hook.f.	Chen et al. 2007	accepted by WFO (placed as the accepted name of a taxon)
175	*Impatiens pseudoviola* Gilg	Song et al. 2022	accepted by WFO (placed as the accepted name of a taxon)
176	*Impatiens psittacina* Hook.f.	Utami and Shimizu 2005	accepted by WFO (placed as the accepted name of a taxon)
177	*Impatiens pterosepala* Hook.f.	Cai et al. 2013	accepted by WFO (placed as the accepted name of a taxon)
178	*Impatiens pterosepala* Hook.f.	Song et al. 2022	accepted by WFO (placed as the accepted name of a taxon)
179	*Impatiens purpurea* Hand.-Mazz.	Song et al. 2005	accepted by WFO (placed as the accepted name of a taxon)
180	*Impatiens purpureifolia* S.H.Huang & Y.M.Shui	Shui et al. 2011	accepted by WFO (placed as the accepted name of a taxon)
181	*Impatiens purpureoviolacea* Gilg	Fischer et al. 2021	accepted by WFO (placed as the accepted name of a taxon)
182	*Impatiens quadriloba* K.M.Liu & Y.L.Xiang	Cai et al. 2013	accepted by WFO (placed as the accepted name of a taxon)
183	*Impatiens quadriloba* K.M.Liu & Y.L.Xiang	Cong et al. 2010	accepted by WFO (placed as the accepted name of a taxon)
184	*Impatiens racemosa* DC.	Zhang et al. 2016	accepted by WFO (placed as the accepted name of a taxon)
185	*Impatiens radiata* Hook.f.	Zhang et al. 2016	accepted by WFO (placed as the accepted name of a taxon)
186	*Impatiens rapiformi***s** Y.Y.Cong & Y.X.Song	Song et al. 2021b	accepted by WFO (placed as the accepted name of a taxon)
187	*Impatiens rapiformis* Y.Y.Cong & Y.X.Song	Song et al. 2022	accepted by WFO (placed as the accepted name of a taxon)
188	*Impatiens rectangula* Hand.-Mazz.	Zhang et al. 2016	accepted by WFO (placed as the accepted name of a taxon)
189	*Impatiens reptans* Hook.f.	Chen et al. 2007	synonym of *Impatiens procumbens* Franch.; accepted name in the genus *Impatiens*
190	*Impatiens rhombifolia* Y.Q.Lu & Y.L.Chen	Lu and Chen 1991	synonym of *Impatiens procumbens* Franch.; accepted name in the genus *Impatiens*
191	*Impatiens rostellata* Franch.	Lu and Chen 1991	accepted by WFO (placed as the accepted name of a taxon)
192	*Impatiens rostellata* Franch.	Song et al. 2005	accepted by WFO (placed as the accepted name of a taxon)
193	*Impatiens rubro-striata* Hook.f.	Cai et al. 2013	accepted by WFO (placed as the accepted name of a taxon)
194	*Impatiens rubro-striata* Hook.f.	Song et al. 2022	accepted by WFO (placed as the accepted name of a taxon)
195	*Impatiens rugata* S.H.Huang & Y.M.Shui	Shui et al. 2011	accepted by WFO (placed as the accepted name of a taxon)
196	*Impatiens ruiliensis* S.Akiyama & H.Ohba	Song et al. 2005	accepted by WFO (placed as the accepted name of a taxon)
197	*Impatiens ruiliensis* S.Akiyama & H.Ohba	Zhang et al. 2016	accepted by WFO (placed as the accepted name of a taxon)
198	*Impatiens rupestris* K.M.Liu & X.Z.Cai	Cai et al. 2013	accepted by WFO (placed as the accepted name of a taxon)
199	*Impatiens rupestris* K.M.Liu & X.Z.Cai	Song et al. 2022	accepted by WFO (placed as the accepted name of a taxon)
200	*Impatiens scabrida* DC.	Song et al. 2005	accepted by WFO (placed as the accepted name of a taxon)
201	*Impatiens scabrida* DC.	Abid et al. 2011	accepted by WFO (placed as the accepted name of a taxon)
202	*Impatiens scabrida* DC.	Zhang et al. 2016	accepted by WFO (placed as the accepted name of a taxon)
203	*Impatiens scullyi* Hook.f.	Zhang et al. 2016	accepted by WFO (placed as the accepted name of a taxon)
204	*Impatiens shennongensis* Qiang Wang & H.P.Deng	Wang et al. 2016	accepted by WFO (placed as the accepted name of a taxon)
205	*Impatiens siamensis* T.Shimizu	Utami and Shimizu 2005	accepted by WFO (placed as the accepted name of a taxon)
206	? *Impatiens siculifer* Hook.f.	Song et al. 2005	Found on WFO as *Impatiens siculifera*, but originally named as *Impatiens siculifer* by Hooker (1908)
207	? *Impatiens siculifer* Hook.f.	Zhang et al. 2016	Found on WFO as *Impatiens siculifera*, but originally named as *Impatiens siculifer* by Hooker (1908)
208	*Impatiens siculifera* Hook.f.	Chen et al. 2007	accepted by WFO (placed as the accepted name of a taxon)
209	? *Impatiens siculifera var. porphyrea* Hook.f.	Chen et al. 2007	accepted by WFO (placed as the accepted name of an infraspecific taxon of the species *Impatiens siculifera* Hook.f.)
210	? *Impatiens siculifera var. mitis* Lingelsh. & Borza	Song et al. 2022	accepted by WFO (placed as the accepted name of an infraspecific taxon of the species *Impatiens siculifera* Hook.f.)
211	*Impatiens sikaiensis* Q. Luo & Ying Yuan	Yuan et al. 2022	accepted by WFO (placed as the accepted name of a taxon)
212	*Impatiens soulieana* Hook.f.	Song et al. 2005	accepted by WFO (placed as the accepted name of a taxon)
213	*Impatiens spirifera* Hook.f. & Thomson	Rewicz et al. 2020a	accepted by WFO (placed as the accepted name of a taxon)
214	*Impatiens stenantha* Hook.f.	Song et al. 2022	accepted by WFO (placed as the accepted name of a taxon)
215	*Impatiens stenosepala* E.Pritz.	Chen et al. 2007	accepted by WFO (placed as the accepted name of a taxon)
216	*Impatiens sterilis* Y.Y. Cong & Y.X.Song	Song et al. 2021a	accepted by WFO (placed as the accepted name of a taxon)
217	*Impatiens stocksii* Hook.f. & Thomson	Utami and Shimizu 2005	accepted by WFO (placed as the accepted name of a taxon)
218	*Impatiens stuhlmannii* Warb.	Song et al. 2022	accepted by WFO (placed as the accepted name of a taxon)
219	*Impatiens sulcata* Wall.	Cai et al. 2013	accepted by WFO (placed as the accepted name of a taxon)
220	*Impatiens sulcata* Wall.	Song et al. 2022	accepted by WFO (placed as the accepted name of a taxon)
221	*Impatiens sulcata* Wall.	Abid et al. 2011	accepted by WFO (placed as the accepted name of a taxon)
222	*Impatiens sulcata* Wall.	Zhang et al. 2016	accepted by WFO (placed as the accepted name of a taxon)
223	*Impatiens sulcata* Wall.	Rewicz et al. 2020a	accepted by WFO (placed as the accepted name of a taxon)
224	*Impatiens sunkoshiensis* S.Akiyama, H.Ohba & Wakab.	Zhang et al. 2016	synonym of *Impatiens laxiflora* Edgew.; accepted name in the genus *Impatiens*
225	*Impatiens sutchuenensis* Franch. ex Hook.f.	Cai et al. 2013	accepted by WFO (placed as the accepted name of a taxon)
226	*Impatiens teitensis* sub. *teitensis*	Song et al. 2022	accepted by WFO (placed as the accepted name of an infraspecific taxon of the species *Impatiens teitensis* Grey-Wilson)
227	*Impatiens textori* Miq.	Utami and Shimizu 2005	accepted by WFO (placed as the accepted name of a taxon)
228	*Impatiens thomsonii* Hook.f.	Abid et al. 2011	accepted by WFO (placed as the accepted name of a taxon)
229	*Impatiens tomentella* Hook.f.	Song et al. 2022	accepted by WFO (placed as the accepted name of a taxon)
230	*Impatiens tongbiguanensis* S.Akiyama & H.Ohba	Cai et al. 2013	synonym of *Impatiens leptoceras* DC.; accepted name in the genus *Impatiens*
231	*Impatiens tortisepala* Hook.f.	Luo et al. 2014	accepted by WFO (placed as the accepted name of a taxon)
232	*Impatiens tripetala* Roxb. & DC.	Song et al. 2022	accepted by WFO (placed as the accepted name of a taxon)
233	*Impatiens troupinii* Eb.Fisch., Abrah., Holstein & S.B.Janssens	Fischer et al. 2021	accepted by WFO (placed as the accepted name of a taxon)
234	*Impatiens tsangshanensis* Y.L.Chen	Song et al. 2022	accepted by WFO (placed as the accepted name of a taxon)
235	*Impatiens tuberculata* Hook.f. & Thomson	Zhang et al. 2016	accepted by WFO (placed as the accepted name of a taxon)
236	*Impatiens uliginosa* Franch.	Song et al. 2022	accepted by WFO (placed as the accepted name of a taxon)
237	*Impatiens undulata* Y.L.Chen & Y.Q.Lu	Lu and Chen 1991	accepted by WFO (placed as the accepted name of a taxon)
238	*Impatiens unguiculata* K.M.Liu & Y.Y.Cong	Cong et al. 2013	accepted by WFO (placed as the accepted name of a taxon)
239	*Impatiens urticifolia* Wall.	Zhang et al. 2016	accepted by WFO (placed as the accepted name of a taxon)
240	*Impatiens urundiensis* Gilg	Fischer et al. 2021	synonym of *Impatiens purpureoviolacea* Gilg.; accepted name in the genus *Impatiens*
241	*Impatiens versicolor* Eb. Fisch., Abrah., Holstein & S.B.Janssens	Fischer et al. 2021	accepted by WFO (placed as the accepted name of a taxon)
242	*Impatiens walleriana* Hook.f.	Song et al. 2005	accepted by WFO (placed as the accepted name of a taxon)
243	*Impatiens walleriana* Hook.f.	Utami and Shimizu 2005	accepted by WFO (placed as the accepted name of a taxon)
244	*Impatiens wawuensis* Bo Ding & S.X.Yu	Ding et al. 2016	accepted by WFO (placed as the accepted name of a taxon)
245	*Impatiens wenshanensis* S.H.Huang	Song et al. 2022	accepted by WFO (placed as the accepted name of a taxon)
246	*Impatiens wilsonii* Hook.f.	Lu and Chen 1991	accepted by WFO (placed as the accepted name of a taxon)
247	*Impatiens wuerstenii* S.B. Janssens & Dessein	Janssens et al. 2018	accepted by WFO (placed as the accepted name of a taxon)
248	*Impatiens wuyiensis* J.S.Wang, Y.F.Lu & X.F.Jin	Wang et al. 2020	accepted by WFO (placed as the accepted name of a taxon)
249	*Impatiens wuyuanensis* Y.L.Chen	Cai et al. 2013	accepted by WFO (placed as the accepted name of a taxon)
250	*Impatiens xanthina* H.F.Comber	Song et al. 2022	accepted by WFO (placed as the accepted name of a taxon)
251	*Impatiens xanthina* H.F.Comber	Song et al. 2005	accepted by WFO (placed as the accepted name of a taxon)
252	*Impatiens xanthina* H.F.Comber	Chen et al. 2007	accepted by WFO (placed as the accepted name of a taxon)
253	*Impatiens xanthina* H.F.Comber	Cong et al. 2008	accepted by WFO (placed as the accepted name of a taxon)
254	*Impatiens xanthinoides* G.W.Hu	Cai et al. 2015	accepted by WFO (placed as the accepted name of a taxon)
255	^*Impatiens xishuangbannaensis* S.H.Huang	Song et al. 2022	unplaced by WFO (a taxonomist hasn’t yet placed the name in the taxonomy), found on IPNI
256	*Impatiens yilingiana* X.F.Jin, Shu Z.Yang & L.Qian	Jin et al. 2008	accepted by WFO (placed as the accepted name of a taxon)
257	*Impatiens yingjiangensis* S.Akiyama & H.Ohba	Cai et al. 2013	accepted by WFO (placed as the accepted name of a taxon)
258	*Impatiens yunlingensis* S.X.Yo, Chang Y.Xia & J.H.Yu	Yu et al. 2022	accepted by WFO (placed as the accepted name of a taxon)
259	^*Impatiens zhaojueensis* Q.Luo	Luo et al. 2023	unplaced by WFO (a taxonomist hasn’t yet placed the name in the taxonomy), found on IPNI

^ - *s*pecies name unchecked and awaiting taxonomic scrutiny by WFO ? - *s*pecies name not found in WFO

**Figure 1.**
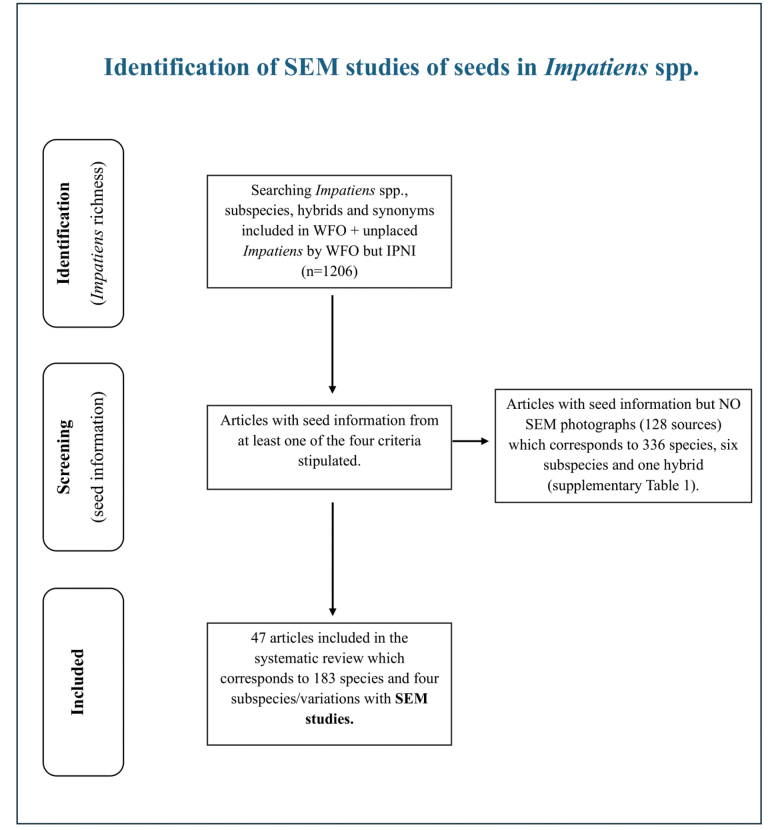
PRISMA flow chart – visualization of the systematic review process, from the initial literature search to the final inclusion of data in studies.

We utilized the PRISMA Flowchart as a visual tool to outline the systematic review process, from the initial literature search to the final inclusion of studies (Page et al. 2021https://estech.shinyapps.io/prisma_flowdiagram/) (Fig. 1).

The species nomenclature validation was adopted from The World Flora Online (WFO 2024), Plants of the World Online (POWO 2024) and International Plant Names Index (IPNI 2024), especially in the most recent publications.

In Table 1, species names are divided into four categories: 1) names confirmed in The World Flora Online (WFO) database – Accepted nomenclature; 2) names that have not yet been confirmed in The World Flora Online (WFO) database – Unchecked and awaiting taxonomic scrutiny; 3) names that appeared under synonyms in the articles - the current names are placed in the column Remarks; 4) species name not found in WFO.

**Figure 2.**
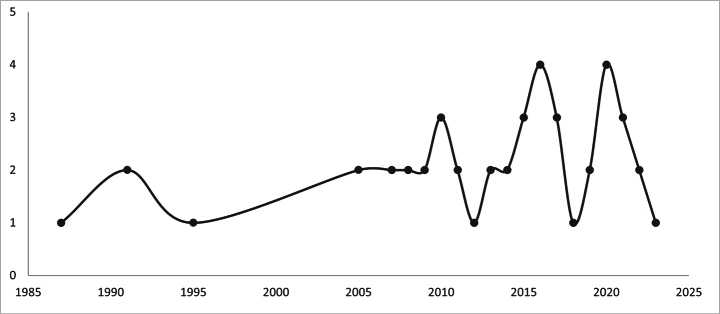
Number of publications featuring SEM-processed seeds for taxa of the genus *Impatiens* (based on data from Table 1).

**Figure 3.**
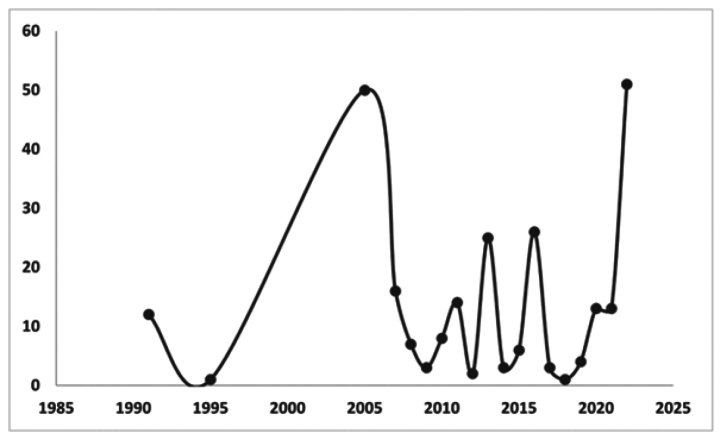
Number of *Impatiens* taxa with SEM-processed seeds over time (based on data from Table 1).

Additionally, three graphs were generated based on three aspects: 1.-number of publications presenting seeds processed with SEM for taxa of the *Impatiens* genus, 2.-number of *Impatiens* taxa with seeds processed with SEM over time, and 3.-number of publications on seeds without SEM photos but with seeds information for taxa of the *Impatiens* genus (Figs 2, 3, 5 respectively)

Finally, to provide a clear visual representation of the countries and species with the highest number of records under the SEM criterion, a heat map was generated in Microsoft Excel 2018 (Fig. 5). Additionally, a word cloud was generated using the “wordcloud” package (Fellows et al. 2018) in R (R Development Core Team 2012) (Fig. 6).

## ﻿Results

### ﻿Literature data with SEM photos

A total of 47 references in the literature pertain to the carpology of seeds within the genus *Impatiens*, including attached SEM images, and encompass information on 183 unique spp. and 4 subspecies/variations (with accepted nomenclature by WFO), (Table 1, Fig. 1).

Single carpological descriptions have been recorded for most species. Only a few species had information available from more than one literature source: for instance, *I. arguta*, *I. balfourii*, and others *I. drepanophora* and others were documented in two sources; *I. aquatilis*, *I. balsamina*, *I. chinensis* in three; and *I. xanthina* in four. In the case of three species (*I. noli-tangere*, *I. pulchra*, and *I. sulcata*), information was found in five publications (Table 1, Fig. 6).

Information regarding the seed carpology of the genus *Impatiens* (with SEM photos) was found in 47 articles, with the earliest ones dating back to the early 1990s. From 1991 to 2004, individual articles on the carpology of the *Impatiens* genus were published sporadically. Subsequently, there was a fluctuating level of publishing activity on this topic, with significant increases in 2016, 2017, 2020, and 2023 (Fig. 2).

Despite the limited number of studies on the carpology of *Impatiens* genus seeds since the early 1990s, notable contributions were made by Song et al. (2005) with 50 taxa, followed by Chen et al. (2007) with 16, and Abid et al. (2011) with 14 taxa. Subsequent significant works providing carpological descriptions for large groups of *Impatiens* taxa included those of Cai et al. (2013) with 25 taxa, Zhang et al. (2016) with 26, and Song et al. (2022) with 51 taxa (Fig. 3).

**Figure 4.**
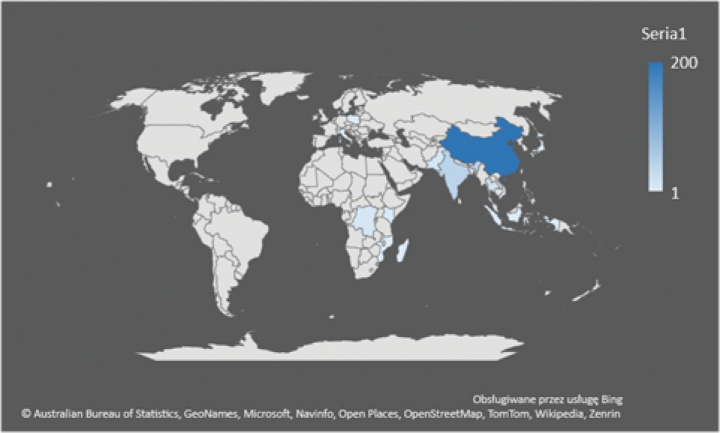
Country of origin of the material of *Impatiens* genus (based on data from Table 1).

Most of the material originated from Asia, particularly from China (187 taxa), India (21), Pakistan (11) and Thailand (9). A few individual samples were sourced from Europe, including Italy (2), Switzerland (2) and Poland (2), but also Japan (2) and Kenya (4) (Fig. 4).

### ﻿Literature data without SEM photos

Through our literature review, we identified around 336 accepted species, six subspecies and one hybrid of seeds belonging to various taxa of the genus *Impatiens*. These descriptions encompassed a range of information, including morphological characteristics, binocular photographs, and illustrations (Suppl. material 1). The publications containing these descriptions do not include SEM photographs.

**Figure 5.**
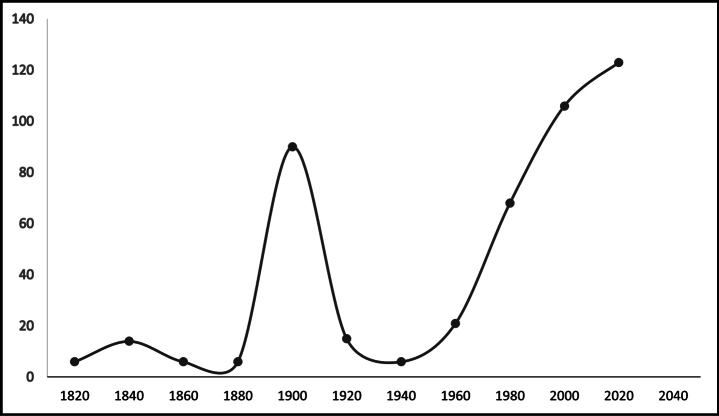
Number of publications and records (online databases) on seeds without SEM photos but with seed information for taxa of the genus *Impatiens* (based on Suppl. material 1).

An analysis of publications on the carpology of the genus *Impatiens* that do not contain SEM images reveals various trends and variability in the number of publications over the past 200 years. During this period, there were 128 publications on this topic. The earliest publications date back to the XIX century (year 1824). The greatest increase in interest occurred after 2000, when articles on specimens of the *Impatiens* genus, most frequently including descriptions of the seeds, began to appear more often (Fig. 5), especially due to the presence of online databases (Suppl. material 1).

**Figure 6.**
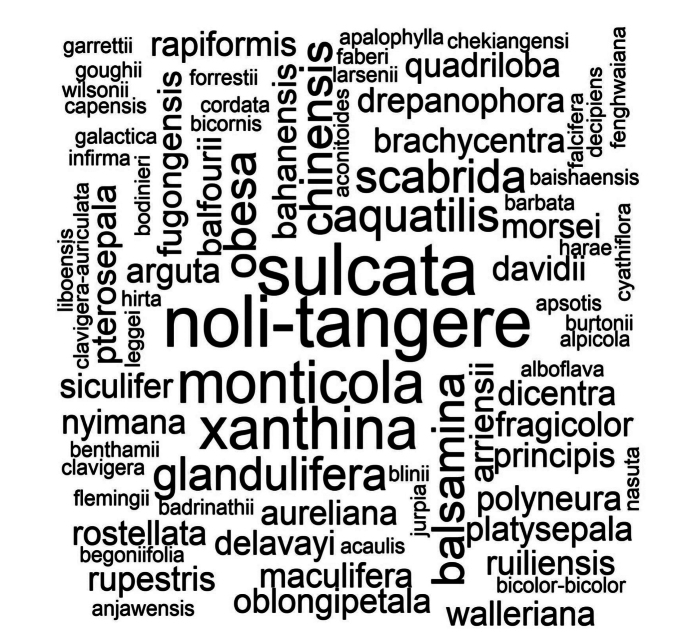
Wordcloud of *Impatiens* species with frequencies of SEM studies. The symbol ‘-’ was added to some species, subspecies and varieties to recognize them as a single word.

## ﻿Discussion and conclusion

Investigating the microstructure of seeds (e. g., primary, and secondary seed coat ornamentation, periclinal cell wall patterns) is essential not only for taxonomic purposes but also as a tool for gaining insights into plant developmental processes, including the adaptation of plants to changing environmental conditions (Utami and Shimizu 2005; Yu et al. 2015; Zhang et al. 2016; Rewicz et al. 2022b). Understanding these adaptations is crucial for comprehending how plants thrive in diverse environments and how they can be protected, particularly in the face of challenges posed by climate change (Bailly and Gomez Roldan 2023). A key element is understanding the micromorphology of seeds and fruits (Van Waveren 2019; Rewicz et al. 2020a; Rewicz et al. 2020b). To facilitate robust conclusions and data comparisons, carpological research, especially regarding seed coat morphology, should be based on high-quality photographs, preferably obtained through scanning electron microscopy. This approach helps mitigate potential discrepancies across different descriptive systems and minimizes researcher subjectivity (Zhao et al. 2023). The introduction of scanning electron microscopy (SEM) was an important moment in scientific advancement, and research using this technology flourished in the late 1990s.

Our analysis of the literature on the microstructure of seeds from the genus *Impatiens* confirms this trend, as the first attempts to use SEM in research on this genus *Impatiens* date back to 1990 (Fig. 2). Unfortunately, despite a significant increase in the number of carpological publications using SEM images, our analysis of literature sources showed that only 21% of taxa from the entire genus, which includes more than 1,000 species, have data with SEM images (Grey-Wilson 1980; Zhao-Cen et al. 2020; Tanaka et al. 2022). The analysis of literature sources also shows that the majority of species are described in only one publication, and the material was collected from a single locality (Table 1). There are, however, exceptions, where several species have garnered more research attention, such as *I. aquatilis*, *I. bahanensis*, *I. balsamina*, *I. chinensis*, *I. fugongensis*, *I. monticola*, *I. noli-tangere*, *I. scabrida*, *I. sulcata*, and *I. xanthina*. However, it’s worth noting that for *Impatiens* species found in Europe, almost all (with the exception of *I. parviflora*) have SEM images of seeds available. This is particularly significant because species like *I. glandulifera* or *I. capensis* are considered invasive. Therefore, any insight into their biology in non-native ranges becomes crucial for effective management and control strategies (Table 1). Our literature analysis revealed that carpological data for over 300 species of *Impatiens* are available, but without descriptions using SEM (Suppl. material 1). At the same time, it should be noted that the seeds of this genus have long been a subject of interest for researchers (Hooker 1910; Fischer 1934).

Our research also revealed a significant oversight in the carpological studies of *Impatiens*, namely the neglect of seed phenotypic variability. Analyzing the variability of quantitative and qualitative characteristics across individuals from diverse locations, especially those covering a wide geographic range, provides valuable insights into the extents of phenotypic variability and the underlying determinants, such as habitat and climate. Such data also serve as a vital resource for understanding the biology and ecology of the studied species (Rewicz et al. 2016; Kostrakiewicz-Gierałt et al. 2022). Furthermore, analyzing phenotypic plasticity enables us to ascertain whether a given structure exhibits stability or substantial intraspecific variability in response to habitat or climatic influences (Stace 1992). Our literature review on *Impatiens* seeds identified only one study, specifically by Rewicz et al. (2020b), that described the phenotypic plasticity of seeds, focusing on an invasive species in Europe - *I. capensis* (Rewicz et al. 2022a).

In our opinion, material availability plays a key role in carpological research. It is noteworthy that research on material from herbarium collections is not always possible due to two main reasons. Firstly, the quantity of material on an herbarium sheet is sometimes limited, rendering it impossible for collection or comprehensive analyses. Secondly, there is a risk of mechanical damage to the material, such as when pressing it into an herbarium sheet, which impedes its utility for research purposes.

Furthermore, it is important to recognize that collecting material from areas of high biodiversity is often restricted for legal reasons. These constraints may pose a significant obstacle in obtaining representative material for carpological research.

The analysis of the literature pertaining to the genus *Impatiens* indicates that majority of the material (seeds) originates from China, accounting for 87% of the cases examined. Following China, the next region with considerably higher seed representation is Madagascar and East-Central Africa. We posit that this distribution pattern is attributed to the abundance of material available from the genus *Impatiens*, as these regions are among the five hot spots with the highest species richness (Grey-Wilson 1980; Yuan et al. 2004; Song 2006; Tabak and von Wettberg 2008; Zika 2009; Ruchisansakun et al. 2014).

Further carpological research on the genus *Impatiens* is imperative for advancing our understanding of these plants. Identifying new species, elucidating their geographical distribution, life cycles, and ecology are among the numerous benefits of such research. Additionally, carpological research can also shed new light on the evolutionary history of these plants and the interrelationships among individual species. Moreover, research on *Impatiens* carpology can be extremely helpful in monitoring the population status of these plants. Given the threats facing many species and their natural habitats, understanding population dynamics is crucial for effective conservation efforts.

In conclusion, carpological research on the genus *Impatiens* is not only of scientific importance, but also of practical significance for the protection of representatives of this genus.

## ﻿Additional information

### Use of AI

No use of AI was reported.

### Author ORCIDs

Agnieszka Rewicz https://orcid.org/0000-0002-8228-6406

René Monzalvo https://orcid.org/0000-0003-1049-5468

Monika Myśliwy https://orcid.org/0000-0001-6573-6455

### Data availability

All of the data that support the results of this study are available in the manuscript and in the Supplementary Information (see Suppl. material 1).

## Supplementary materials

Supplementary material 1 *Impatiens* species with seed information but no SEM photographs Data type docx https://binary.pensoft.net/file/1407456This dataset is made available under the Open Database License (http://opendatacommons.org/licenses/odbl/1.0/). The Open Database License (ODbL) is a license agreement intended to allow users to freely share, modify, and use this Dataset while maintaining this same freedom for others, provided that the original source and author(s) are credited.Agnieszka Rewicz, René Monzalvo, Kayla Grey, Katarzyna Sanek, Maja Mackiewicz, Monika Myśliwy
