# Supplementary material for: ﻿Gap analysis of knowledge about the microstructure of Impatiens Riv. ex L. (Balsaminaceae) seeds using SEM techniques
Source: PhytoKeys. 2025 Sep 2;262:105–28. doi: 10.3897/phytokeys.262.152205 (PMC12421252; doi:10.3897/phytokeys.262.152205)
Supplement: Supplementary material 1 — Impatiens species with seed information but no SEM photographs [file phytokeys-262-105_article-152205__-s001.docx]

**Supplementary Table 1.** *Impatiens* species with seed information but no SEM photographs.

|  |  |  |
| --- | --- | --- |

| **Number** | **Species** | **Morphological description/notes of the seed** | **Photo** | **Diagram** | **Country of origin of the material** | **Authors** | **Publication date** | **Remarks** |
| --- | --- | --- | --- | --- | --- | --- | --- | --- |
| 1 | *Impatiens aadishankarii* Bhaskar & Sringesh | YES | NO | NO | India | India Biodiversity Portal (https://indiabiodiversity.org/species/show/281287) | 2024 | accepted by WFO (placed as the accepted name of a taxon) |
| 2 | [*Impatiens abbatis* Hook.f.](https://www.worldfloraonline.org/taxon/wfo-0001274747) | YES | NO | YES | China | *Hooker | 1908 | accepted by WFO (placed as the accepted name of a taxon) |
| 3 | [*Impatiens abbatis* Hook.f.](https://www.worldfloraonline.org/taxon/wfo-0001274747) | YES | NO | YES | China | Chen et al.  eFloras (Flora of China)  (<http://www.efloras.org/florataxon.aspx?flora_id=2&taxon_id=242445474>) | 2008 | accepted by WFO (placed as the accepted name of a taxon) |
| 4 | *Impatiens adamowskiana* Gogoi & Borah | YES | YES | NO | India | Gogoi and Borah | 2015 | accepted by WFO (placed as the accepted name of a taxon) |
| 5 | *Impatiens agastyamalayensis* (Bhaskar) A.Joe, V.Bhaskar & M.Sabu | YES | YES | NO | India | Joe et al. | 2017 | accepted by WFO (placed as the accepted name of a taxon) |
| 6 | *Impatiens agumbeana* Bhaskar and Razi | YES | NO | YES | India | Bhaskar and Razi | 1982 | accepted by WFO (placed as the accepted name of a taxon) |
| 7 | *Impatiens agumbeana* Bhaskar and Razi | YES | NO | NO | India | India Biodiversity Portal (https://indiabiodiversity.org/species/show/281290) | 2024 | accepted by WFO (placed as the accepted name of a taxon) |
| 8 | *Impatiens aitchisonii* Hook.f. | YES | NO | YES | Afghanistan | **Hooker | 1910 | synonym of ***Impatiens thomsonii*** Hook.f.; accepted name in the genus *Impatiens* |
| 9 | *Impatiens amplexicaulis* Edgew. | YES | NO | NO | India | Edgeworth | 1846 | accepted by WFO (placed as the accepted name of a taxon) |
| 10 | *Impatiens anaimudica* C.E.C. Fish. | YES | NO | YES | India | Biju | 2001 | accepted by WFO (placed as the accepted name of a taxon) |
| 11 | *Impatiens angustisepala* Tardieu | YES | NO | NO | Laos | Tardieu-Blot | 1944 | synonym of ***Impatiens ecalcarata*** Collett & Hemsl.; accepted name in the genus *Impatiens* |
| 12 | *Impatiens anhuiensis* Y.L.Chen | YES | NO | NO | China | Chen | 1999 | accepted by WFO (placed as the accepted name of a taxon) |
| 13 | *Impatiens anhuiensis* Y.L.Chen | YES | NO | NO | China | Chen et al.  eFloras (Flora of China)  (<http://www.efloras.org/florataxon.aspx?flora_id=2&taxon_id=242326669>) | 2008 | accepted by WFO (placed as the accepted name of a taxon) |
| 14 | *Impatiens alboarenicola* Y.Fujimoto, Rabarison & Tagane | YES | YES | NO | Madagascar | Fujimoto et al. | 2024 | Unplaced by WFO (a taxonomist hasn’t yet placed the name in the taxonomy), found on IPNI |
| 15 | *Impatiens aliciae* C.E.C.Fish. | YES | NO | NO | India | Mani et al. | 2020 | accepted by WFO (placed as the accepted name of a taxon) |
| 16 | *Impatiens aliciae* C.E.C.Fish. | YES | NO | NO | India | India Biodiversity Portal  (https://indiabiodiversity.org/species/show/281434) | 2024 | accepted by WFO (placed as the accepted name of a taxon) |
| 17 | *Impatiens aquatica* Bhaskar | YES | NO | NO | India | India Biodiversity Portal  (<https://indiabiodiversity.org/species/show/281293>) | 2024 | accepted by WFO (placed as the accepted name of a taxon |
| 18 | *Impatiens arctosepala* Hook.f. | YES | NO | NO | China | Chen et al.  eFloras (Flora of China)  (http://www.efloras.org/florataxon.aspx?flora_id=2&taxon_id=242445479) | 2008 | accepted by WFO (placed as the accepted name of a taxon |
| 19 | *Impatiens arguta var. Bulleyana* Hook.f. | YES | NO | YES | China | *Hooker | 1908 | synonym of ***Impatiens arguta*** Hook.f. & Thomson.; accepted name in the genus *Impatiens* |
| 20 | *Impatiens arnottii* Thwaites | YES | NO | NO | Sri-Lanka | Thwaites | 1858 | accepted by WFO (placed as the accepted name of a taxon |
| 21 | *Impatiens ashihoi* Gogoi & Borah | YES | YES | NO | India | =Gogoi and Borah | 2015 | accepted by WFO (placed as the accepted name of a taxon) |
| 22 | *Impatiens assurgens* Baker | YES | NO | NO | Africa | Grey-Wilson | 1980 | accepted by WFO (placed as the accepted name of a taxon) |
| 23 | *Impatiens aurella* Rydb | YES | NO | NO | USA | Douglas et al. | 1998 | accepted by WFO (placed as the accepted name of a taxon) |
| 24 | *Impatiens auriculata* Wight | YES | NO | YES | India | Wight | 1837 | accepted by WFO (placed as the accepted name of a taxon) |
| 25 | *Impatiens auriculata* Wight | YES | NO | NO | India | India Biodiversity Portal  (https://indiabiodiversity.org/species/show/253738) | 2024 | accepted by WFO (placed as the accepted name of a taxon) |
| 26 | *Impatiens bababudenensis* Hook.f. | YES | NO | NO | India | **Hooker | 1910 | accepted by WFO (placed as the accepted name of a taxon) |
| 27 | *Impatiens banen* Cheek, Darbyshire and Onana | YES | NO | YES | Cameroon | Cheek et al. | 2023 | **not found on WFO, found on IPNI** |
| 28 | *Impatiens baokangensis* Q.L.Gan & X.W.Li | YES | YES | NO | China | Gan and Li | 2016 | accepted by WFO (placed as the accepted name of a taxon) |
| 29 | *Impatiens barberi* Hook.f. | YES | NO | NO | India | India Biodiversity Portal  (https://indiabiodiversity.org/species/show/253730) | 2024 | accepted by WFO (placed as the accepted name of a taxon |
| 30 | *Impatiens bhaskarii* J.R.N. Dessai, L. Joseph & Janarth. | YES | NO | YES | India | Dessai et al. | 2009 | accepted by WFO (placed as the accepted name of a taxon |
| 31 | *Impatiens bhaskarii* J.R.N. Dessai, L. Joseph & Janarth. | YES | NO | NO | India | Indian Biodiversity Portal (https://indiabiodiversity.org/species/show/281296) | 2024 | accepted by WFO (placed as the accepted name of a taxon |
| 32 | *Impatiens bicornuta* Wall | YES | NO | NO | India | Roxburgh | 1824 | accepted by WFO (placed as the accepted name of a taxon |
| 33 | *Impatiens bicornuta* Wall | YES | NO | NO | India | Indian Biodiversity Portal (https://indiabiodiversity.org/species/show/253703) | 2024 | accepted by WFO (placed as the accepted name of a taxon |
| 34 | *Impatiens bijieensis* X.X.Bai & L.Y.Ren | YES | YES | NO | China | Ren et al. | 2022 | accepted by WFO (placed as the accepted name of a taxon) |
| 35 | *Impatiens bisaccata* Warb. | YES | NO | NO | Madagascar | Warburg | 1895 | accepted by WFO (placed as the accepted name of a taxon) |
| 36 | *Impatiens bonii* Hook.f. | YES | NO | YES | Vietnam | *Hooker | 1908 | accepted by WFO (placed as the accepted name of a taxon) |
| 37 | *Impatiens bracteata* Colebr. ex Roxb | YES | NO | NO | India | Indian Biodiversity Portal (https://indiabiodiversity.org/species/show/253676) | 2024 | accepted by WFO (placed as the accepted name of a taxon) |
| 38 | *Impatiens brachycentra* G.M.Schulze & Launert | YES | NO | NO | Malawi | Launert | 1962 | synonym of ***Impatiens polyantha*** Gilg.; accepted name in the genus *Impatiens* |
| 39 | *Impatiens brahmagiriana* Sarav. & Kaliam. | YES | YES | NO | India | Saravanan and Kaliamoorthy | 2024 | Unplaced by WFO (a taxonomist hasn’t yet placed the name in the taxonomy), found on IPNI |
| 40 | *Impatiens brevicornis* (E.Barnes) Bhaskar | YES | NO | NO | India | Indian Biodiversity Portal (https://indiabiodiversity.org/species/show/281300) | 2024 | accepted by WFO (placed as the accepted name of a taxon |
| 41 | *Impatiens brevicornis* (E.Barnes) Bhaskar | YES | NO | NO | India | Prabhukumar et al. | 2018 | accepted by WFO (placed as the accepted name of a taxon |
| 42 | *Impatiens briartii* De Wild. & T.Durand | YES | NO | NO | Africa (country?) | Grey-Wilson | 1980 | accepted by WFO (placed as the accepted name of a taxon |
| 43 | *Impatiens brittoi* B.Mani & S.Thomas | YES | YES | NO | India | Mani and Thomas | 2017 | accepted by WFO (placed as the accepted name of a taxon |
| 44 | *Impatiens burmanica* Hook.f. | YES | NO | NO | Myanmar | Toppin | 1920 | accepted by WFO (placed as the accepted name of a taxon |
| 45 | *Impatiens campanulata* Wight | YES | NO | YES | India | Wight | 1837 | accepted by WFO (placed as the accepted name of a taxon |
| 46 | *Impatiens campanulata* Wight | YES | NO | NO | India | Indian Biodiversity Portal  (https://indiabiodiversity.org/species/show/262989) | 2024 | accepted by WFO (placed as the accepted name of a taxon |
| 47 | *Impatiens capusii* Hook.f. | YES | NO | YES | Vietnam | *Hooker | 1908 | unplaced by WFO (a taxonomist hasn’t yet placed the name in the taxonomy), found on IPNI |
| 48 | *Impatiens cavaleriei* X.X. Bai & R.X.Huang | YES | YES | YES | China | Huang et al. | 2023 | unplaced by WFO (a taxonomist hasn’t yet placed the name in the taxonomy), found on IPNI |
| 49 | *Impatiens cecilii* N.E.Br. | YES | NO | NO | Africa (country?) | Grey-Wilson | 1980 | accepted by WFO (placed as the accepted name of a taxon) |
| 50 | *Impatiens charanii* T. Shimizu | YES | NO | YES | Thailand | Shimizu | 1991 | accepted by WFO (placed as the accepted name of a taxon) |
| 51 | *Impatiens charanii* T. Shimizu | YES | NO | NO | Thailand | Utami and Shimizu | 2005 | accepted by WFO (placed as the accepted name of a taxon) |
| 52 | *Impatiens chapaensis* Tardieu | YES | NO | NO | Vietnam | Tardieu | 1944 | accepted by WFO (placed as the accepted name of a taxon) |
| 53 | *Impatiens chonoceras* Hassk. | YES | NO | NO | Indonesia; Jawa | Hasskarl | 1858 | **unplaced by WFO (a taxonomist hasn’t yet placed the name in the taxonomy)** |
| 54 | *Impatiens chumphonensis* T. Shimizu | YES | NO | YES | Thailand | Shimizu | 2000 | accepted by WFO (placed as the accepted name of a taxon) |
| 55 | *Impatiens chungtienensis* Y.L.Chen | YES | NO | YES | China | Chen | 1978 | accepted by WFO (placed as the accepted name of a taxon |
| 56 | *Impatiens chungtienensis* Y.L.Chen | YES | NO | YES | China | Chen et al.  eFloras of China (Flora of China)  (http://www.efloras.org/florataxon.aspx?flora_id=2&taxon_id=242326675) | 1978 | accepted by WFO (placed as the accepted name of a taxon |
| 57 | *Impatiens clavata* Bhaskar | YES | NO | NO | India | Bhaskar | 2006 | accepted by WFO (placed as the accepted name of a taxon) |
| 58 | *Impatiens clavata* Bhaskar | YES | NO | NO | India | India Biodiversity Portal  ( <https://indiabiodiversity.org/species/show/281304>) | 2024 | accepted by WFO (placed as the accepted name of a taxon) |
| 59 | *Impatiens clavicornu* Turcz. | YES | NO | NO | India | India Biodiversity Portal  (https://indiabiodiversity.org/species/show/253700) | 2024 | accepted by WFO (placed as the accepted name of a taxon) |
| 60 | *Impatiens clavicuspis* Hook.f. ex W.W.Sm. | YES | NO | NO | China | Smith | 1915 | accepted by WFO (placed as the accepted name of a taxon) |
| 61 | *Impatiens clavicuspis* Hook.f. ex W.W.Sm. | YES | NO | NO | China | Chen et al.  eFloras of China (Flora of China)  (http://www.efloras.org/florataxon.aspx?flora_id=2&taxon_id=242326676) | 2008 | accepted by WFO (placed as the accepted name of a taxon) |
| 62 | *Impatiens coelotropis* C.E.C.Fisch. | YES | NO | NO | India | India Biodiversity Portal  (https://indiabiodiversity.org/species/show/253694) | 2024 | accepted by WFO (placed as the accepted name of a taxon) |
| 63 | *Impatiens compta* Hook.f. | YES | NO | NO | China | Chen et al.  eFloras of China (Flora of China)  (http://www.efloras.org/florataxon.aspx?flora_id=2&taxon_id=242445495) | 2008 | accepted by WFO (placed as the accepted name of a taxon) |
| 64 | *Impatiens conaensis* Y.L.Chen | YES | NO | NO | China | Chen et al.  eFloras (Flora of China)  (http://www.efloras.org/florataxon.aspx?flora_id=2&taxon_id=242326677) | 2008 | accepted by WFO (placed as the accepted name of a taxon) |
| 65 | *Impatiens concinna* Hook.f. | YES | NO | NO | India | Hooker | 1874 | accepted by WFO (placed as the accepted name of a taxon) |
| 66 | *Impatiens concinna* Hook.f. | YES | NO | NO | India | India Biodiversity Portal  (https://indiabiodiversity.org/species/show/262992) | 2024 | accepted by WFO (placed as the accepted name of a taxon) |
| 67 | *Impatiens courtallensis* Ramas. & Pandur*.* | YES | NO | YES | India | Ramasubu et al. | 2015 | accepted by WFO (placed as the accepted name of a taxon) |
| 68 | *Impatiens courtallensis* Ramas. & Pandur*.* | YES | NO | NO | India | Flowers of India  (https://www.flowersofindia.net/catalog/slides/Courtallam%20Balsam.html) | 2015 | accepted by WFO (placed as the accepted name of a taxon) |
| 69 | *Impatiens corchorifolia* Franch | YES | NO | NO | China | ***Hooker | 1908 | accepted by WFO (placed as the accepted name of a taxon) |
| 70 | *Impatiens cothurnoides* C.E.C.Fisch. | YES | NO | NO | India | Fischer | 1931 | accepted by WFO (placed as the accepted name of a taxon) |
| 71 | *Impatiens cothurnoides* C.E.C.Fisch. | YES | NO | NO | India | India Biodiversity Portal  (https://indiabiodiversity.org/species/show/253758) | 2008 | accepted by WFO (placed as the accepted name of a taxon) |
| 72 | *Impatiens crassiloba* Hook.f. | YES | NO | NO | China | ***Hooker | 1908 | synonym of ***Impatiens procumbens*** Franch.; accepted name in the genus *Impatiens* |
| 73 | *Impatiens crenata* Bedd. | YES | NO | NO | India | India Biodiversity Portal  (https://indiabiodiversity.org/species/show/264378 | 2024 | accepted by WFO (placed as the accepted name of a taxon |
| 74 | *Impatiens cuspidata* Wight & Arn. | YES | NO | NO | India | Indian Biodiversity Portal (https://indiabiodiversity.org/observation/show/16107701) | 2024 | accepted by WFO (placed as the accepted name of a taxon) |
| 75 | *Impatiens dalaiensis* Gogoi & Borah | YES | YES | NO | India | %%Gogoi and Borah | 2015 | accepted by WFO (placed as the accepted name of a taxon) |
| 76 | *Impatiens dasysperma* Wight | YES | NO | YES | India | Wight | 1837 | accepted by WFO (placed as the accepted name of a taxon) |
| 77 | *Impatiens dasysperma* Wight | YES | NO | NO | India | Indian Biodiversity Portal (https://indiabiodiversity.org/species/show/253732) | 2024 | accepted by WFO (placed as the accepted name of a taxon) |
| 78 | *Impatiens debilis* Turcz. | YES | NO | YES | India | ****Hooker | 1910 | accepted by WFO (placed as the accepted name of a taxon) |
| 79 | *Impatiens delicatula* Baill. | NO | NO | YES | Madagascar | Baillon | 1891 | accepted by WFO (placed as the accepted name of a taxon) |
| 80 | *Impatiens densifolia* G.M.Schulze & Wilczek | YES | NO | NO | Zaire | Grey-Wilson | 1980 | accepted by WFO (placed as the accepted name of a taxon) |
| 82 | *Impatiens desmantha* Hook.f. | YES | NO | NO | China | ***Hooker | 1908 | accepted by WFO (placed as the accepted name of a taxon) |
| 83 | *Impatiens desmantha* Hook.f. | YES | NO | NO | China | Chen et al.  eFloras (Flora of China)  (http://www.efloras.org/florataxon.aspx?flora_id=2&taxon_id=242445503) | 2008 | accepted by WFO (placed as the accepted name of a taxon) |
| 84 | *Impatiens devolii* T.C.Huang | YES | NO | YES | China | Huang | 1973 | accepted by WFO (placed as the accepted name of a taxon) |
| 85 | *Impatiens devolii* T.C.Huang | YES | NO | NO | China | Chen et al.  eFloras of China (Flora of China)  (http://www.efloras.org/florataxon.aspx?flora_id=2&taxon_id=242413426) | 2008 | accepted by WFO (placed as the accepted name of a taxon) |
| 86 | *Impatiens diffusa* Hook.f. | YES | NO | NO | Indo-China | India Biodiversity Portal  (https://indiabiodiversity.org/species/list?max=16&offset=0&sort=species.lastUpdated&userGroupList&view=grid) | 1909 | accepted by WFO (placed as the accepted name of a taxon) |
| 87 | *Impatiens diffusa* Hook.f. | YES | NO | YES | Cambodia | ****Hooker | 1910 | accepted by WFO (placed as the accepted name of a taxon) |
| 88 | *Impatiens dindigulensis* Ramas., Anjana & Chandra Prabha | YES | YES | NO | India | Ramasubbu et al. | 2020 | accepted by WFO (placed as the accepted name of a taxon) |
| 89 | *Impatiens disotis* Hook.f. | YES | NO | NO | India | India Biodiversity Portal  (https://indiabiodiversity.org/species/show/262994) | 2024 | accepted by WFO (placed as the accepted name of a taxon) |
| 90 | *Impatiens distracta* Hook.f. | YES | NO | NO | China | **Hooker | 1910 | accepted by WFO (placed as the accepted name of a taxon) |
| 91 | *Impatiens distracta* Hook.f. | YES | NO | NO | China | Chen et al.  eFloras (Flora of China)  (http://www.efloras.org/florataxon.aspx?flora_id=2&taxon_id=250076983) | 2008 | accepted by WFO (placed as the accepted name of a taxon) |
| 92 | *Impatiens divaricata* Franch*.* | YES | NO | NO | China | Franchet | 1886 | accepted by WFO (placed as the accepted name of a taxon) |
| 93 | *Impatiens divaricata* Franch. | YES | NO | NO | China | ***Hooker | 1908 | accepted by WFO (placed as the accepted name of a taxon) |
| 94 | *Impatiens divaricata* Franch. | YES | NO | NO | China | Chen et al.  eFloras (Flora of China)  (http://www.efloras.org/florataxon.aspx?flora_id=2&taxon_id=242445509) | 2008 | accepted by WFO (placed as the accepted name of a taxon) |
| 95 | *Impatiens duclouxii* Hook.f. | YES | NO | NO | China | ***Hooker | 1908 | accepted by WFO (placed as the accepted name of a taxon) |
| 96 | *Impatiens duclouxii* Hook.f. | YES | NO | NO | China | Chen et al.  eFloras (Flora of China)  (http://www.efloras.org/florataxon.aspx?flora_id=2&taxon_id=242445510) | 2008 | accepted by WFO (placed as the accepted name of a taxon) |
| 97 | *Impatiens ecornuta* Gerry Moore, Zika & Rushworth | YES | NO | NO | USA | Douglas et al. | 1998 | accepted by WFO (placed as the accepted name of a taxon) |
| 98 | *Impatiens elachistocentra* G.M.Schulze ex Schlieben | YES | NO | NO | Tanzania | Grey-Wilson | 1980 | accepted by WFO (placed as the accepted name of a taxon) |
| 99 | *Impatiens elegans* Bedd. | YES | NO | NO | India | Beddome | 1859 | accepted by WFO (placed as the accepted name of a taxon) |
| 100 | *Impatiens elianae* Abrah. & Eb.Fisch*.* | YES | NO | NO | Madagascar | Abrahamcyk et al. | 2015 | accepted by WFO (placed as the accepted name of a taxon) |
| 101 | *Impatiens erubescens* Dunn | YES | NO | NO | Myanmar | Toppin | 1920 | accepted by WFO (placed as the accepted name of a taxon) |
| 102 | *Impatiens eryaleia* Launert | YES | NO | NO | Malawi | Launert | 1962 | accepted by WFO (placed as the accepted name of a taxon) |
| 103 | *Impatiens eryaleia* Launert | YES | NO | NO | Africa (country?) | Grey-Wilson | 1980 | accepted by WFO (placed as the accepted name of a taxon) |
| 104 | *Impatiens ethiopica* Grey-Wilson | YES | NO | NO | Africa (country?) | Grey-Wilson | 1980 | accepted by WFO (placed as the accepted name of a taxon) |
| 105 | *Impatiens etugei* Cheek, Darbyshire and Onana | YES | NO | YES | Cameroon | Cheek et al. | 2023 | not found on WFO, found on IPNI |
| 106 | *Impatiens exiguiflora* Hook.f. | YES | NO | YES | China | *****Hooker | 1911 | accepted by WFO (placed as the accepted name of a taxon) |
| 107 | *Impatiens exiguiflora* Hook.f. | YES | NO | YES | China | Chen et al.  eFloras (Flora of China)  (http://www.efloras.org/florataxon.aspx?flora_id=2&taxon_id=242445512) | 2008 | accepted by WFO (placed as the accepted name of a taxon) |
| 108 | *Impatiens exilis* Hook.f. | NO | YES | NO | Nepal | Raskoti and Ale | 2022 | accepted by WFO (placed as the accepted name of a taxon) |
| 109 | *Impatiens fargesii* Hook.f. | YES | NO | NO | China | ***Hooker | 1908 | accepted by WFO (placed as the accepted name of a taxon) |
| 110 | *Impatiens fargesii* Hook.f. | YES | NO | NO | China | Chen et al.  eFloras (Flora of China)  (http://www.efloras.org/florataxon.aspx?flora_id=2&taxon_id=242445515) | 2008 | accepted by WFO (placed as the accepted name of a taxon) |
| 111 | *Impatiens fianarantsoae* Eb.Fisch. & Raheliv. | YES | NO | NO | Madagascar | Fischer and Rahelivololona | 2007 | accepted by WFO (placed as the accepted name of a taxon) |
| 112 | *Impatiens filicaulis* Hook.f. | YES | NO | NO | Philippines | ******Hooker | 1909 | accepted by WFO (placed as the accepted name of a taxon) |
| 113 | *Impatiens filicornu* Hook.f. | YES | NO | NO | Africa (country?) | Grey-Wilson | 1980 | accepted by WFO (placed as the accepted name of a taxon) |
| 114 | *Impatiens flaccida* Arn. | YES | NO | NO | India | India Biodiversity Portal  (https://indiabiodiversity.org/species/show/230014) | 2024 | accepted by WFO (placed as the accepted name of a taxon) |
| 115 | *Impatiens flemingii* Hook.f. | YES | NO | NO | India | India Biodiversity Portal  (https://indiabiodiversity.org/species/show/281315) | 2024 | accepted by WFO (placed as the accepted name of a taxon) |
| 116 | *Impatiens floribunda* Wight | YES | NO | NO | India | India Biodiversity Portal  (https://indiabiodiversity.org/species/show/253712) | 2024 | accepted by WFO (placed as the accepted name of a taxon) |
| 117 | *Impatiens formosa* Hook.f. | YES | NO | NO | India | India Biodiversity Portal  (https://indiabiodiversity.org/species/show/281317) | 2024 | accepted by WFO (placed as the accepted name of a taxon) |
| 118 | *Impatiens frithii* Cheek | YES | NO | YES | Cameroon | Cheek and Csiba | 2002 | accepted by WFO (placed as the accepted name of a taxon) |
| 119 | *Impatiens fruticosa* Lesch. ex DC. | YES | NO | NO | India | India Biodiversity Portal  (https://indiabiodiversity.org/species/show/262995) | 2024 | accepted by WFO (placed as the accepted name of a taxon) |
| 120 | *Impatiens gamblei* Hook.f. | YES | NO | NO | India | India Biodiversity Portal  (https://indiabiodiversity.org/species/show/281318) | 2024 | accepted by WFO (placed as the accepted name of a taxon) |
| 121 | *Impatiens ganpiuana* Hook.f. | YES | NO | NO | China | *Hooker | 1908 | synonym of ***Impatiens procumbens*** Franch.; accepted name in the genus *Impatiens* |
| 122 | *Impatiens ganpiuana* Hook.f. | YES | NO | NO | China | Chen et al.  eFloras (Flora of China)  (http://www.efloras.org/florataxon.aspx?flora_id=2&taxon_id=242445517) | 2008 | synonym of ***Impatiens procumbens*** Franch.; accepted name in the genus *Impatiens* |
| 123 | *Impatiens gasterocheila* Hook.f. | YES | NO | NO | China | **Hooker | 1910 | accepted by WFO (placed as the accepted name of a taxon) |
| 124 | *Impatiens gasterocheila* Hook.f. | YES | NO | NO | China | Chen et al.  eFloras (Flora of China)  (http://www.efloras.org/florataxon.aspx?flora_id=2&taxon_id=242445518) | 2008 | accepted by WFO (placed as the accepted name of a taxon) |
| 125 | *Impatiens glabrata* K.M.P.Kumar, Hareesh & Bhaskar | YES | YES | NO | India | Prabhukumar et al. | 2016 | accepted by WFO (placed as the accepted name of a taxon) |
| 126 | *Impatiens glabrata* K.M.P.Kumar, Hareesh & Bhaskar | YES | NO | NO | India | India Biodiversity Portal  (https://indiabiodiversity.org/species/show/281321) | 2024 | accepted by WFO (placed as the accepted name of a taxon) |
| 127 | *Impatiens glandulosa* Tardieu | YES | NO | NO | Laos | Tardieu-Blot | 1944 | unplaced by WFO (a taxonomist hasn’t yet placed the name in the taxonomy), found on IPNI |
| 128 | *Impatiens glauca* Hook.f. & Thomson | YES | NO | NO | India | Hook and Thomson | 1859 | accepted by WFO (placed as the accepted name of a taxon) |
| 129 | *Impatiens glauca* Hook.f. & Thomson | YES | NO | NO | India | India Biodiversity Portal  (https://indiabiodiversity.org/species/show/253759) | 1859 | accepted by WFO (placed as the accepted name of a taxon) |
| 130 | *Impatiens godfreyi* Richard, Karuppusamy and Ravichandran | YES | YES | YES | India | Richard et al. | 2021 | unplaced by WFO (a taxonomist hasn’t yet placed the name in the taxonomy), found on IPNI |
| 131 | *Impatiens gomphophylla* Baker | YES | NO | NO | Africa (country?) | Grey-Wilson | 1980 | accepted by WFO (placed as the accepted name of a taxon) |
| 132 | *Impatiens gongolana* N.Hallé | YES | NO | NO | Africa (country?) | Grey-Wilson | 1980 | accepted by WFO (placed as the accepted name of a taxon) |
| 133 | *Impatiens gossweileri* G.M.Schulze | YES | NO | NO | Africa (country?) | Grey-Wilson | 1980 | accepted by WFO (placed as the accepted name of a taxon) |
| 134 | *Impatiens gracilipes* Hook.f. | YES | NO | YES | China | *****Hooker | 1911 | synonym of ***Impatiens desmantha*** Hook.f.; accepted name in the genus *Impatiens* |
| 135 | *Impatiens gracilipes* Hook.f. | YES | NO | YES | China | Chen et al.  eFloras (Flora of China)  (http://www.efloras.org/florataxon.aspx?flora_id=2&taxon_id=242445520) | 1911 | synonym of ***Impatiens desmantha*** Hook.f.; accepted name in the genus *Impatiens* |
| 136 | *Impatiens grandis* B.Heyne | YES | NO | NO | India | Wight | 1837 | accepted by WFO (placed as the accepted name of a taxon) |
| 137 | *Impatiens grandis* B.Heyne | YES | NO | NO | India | India Biodiversity Portal  (https://indiabiodiversity.org/species/show/253745) | 2024 | accepted by WFO (placed as the accepted name of a taxon) |
| 138 | *Impatiens hamata* Warb. | YES | NO | NO | Tanzania | Grey-Wilson | 1980 | accepted by WFO (placed as the accepted name of a taxon) |
| 139 | *Impatiens hambaeksanensis* B.U.Oh | YES | YES | NO | Korea | Oh et al. | 2022 | accepted by WFO (placed as the accepted name of a taxon) |
| 140 | *Impatiens haridasanii* Hareesh & M.Sabu | YES | NO | NO | India | Rewicz et al. | 2020 | accepted by WFO (placed as the accepted name of a taxon) |
| 141 | *Impatiens harmandi* (original description name) Hooker.f. | YES | NO | YES | Vietnam | *Hooker | 1908 | accepted by WFO (placed as the accepted name of a taxon) |
| 142 | *Impatiens hochstetteri* Warb. | YES | NO | NO | Africa (country) | Grey-Wilson | 1980 | accepted by WFO (placed as the accepted name of a taxon) |
| 143 | *Impatiens henanensis* Y.L.Chen | YES | NO | NO | China | Chen | 1999 | accepted by WFO (placed as the accepted name of a taxon) |
| 144 | *Impatiens herbicola* Hook.f. | YES | NO | NO | India | *******Hooker | 1911 | accepted by WFO (placed as the accepted name of a taxon) |
| 145 | *Impatiens herbicola* Hook.f. | YES | NO | NO | India | India Biodiversity Portal (https://indiabiodiversity.org/species/show/262998) | 2024 | accepted by WFO (placed as the accepted name of a taxon) |
| 146 | *Impatiens hians* Hook.f. | YES | NO | NO | Africa (country?) | Grey-Wilson | 1980 | accepted by WFO (placed as the accepted name of a taxon) |
| 147 | *Impatiens hobsonii* Hook.f. | YES | NO | NO | India | India Biodiversity Portal  (https://indiabiodiversity.org/species/show/281325) | 2024 | accepted by WFO (placed as the accepted name of a taxon) |
| 148 | *Impatiens hochstetteri* Warb. | YES | NO | NO | Africa (country?) | Grey-Wilson | 1980 | accepted by WFO (placed as the accepted name of a taxon) |
| 149 | *Impatiens hoehnelii* T.C.E.Fr. | YES | NO | NO | Kenya | Grey-Wilson | 1980 | accepted by WFO (placed as the accepted name of a taxon) |
| 150 | *Impatiens hongsonensis* T.Shimizu | YES | NO | NO | Thailand | Utami and Shimizu | 2005 | accepted by WFO (placed as the accepted name of a taxon) |
| 151 | *Impatiens horizontalis* Latt, B.B.Park & Nob.Tanaka | YES | YES | NO | Myanmar | Latt et al. | 2023 | unplaced by WFO (a taxonomist hasn’t yet placed the name in the taxonomy), found on IPNI |
| 152 | *Impatiens hubertti* Hook.f. | YES | NO | NO | Borneo | %Hooker | 1910 | unplaced by WFO (a taxonomist hasn’t yet placed the name in the taxonomy), found on IPNI |
| 153 | *Impatiens humblotiana* Baill. | NO | NO | YES | Madagascar | Baillon | 1891 | accepted by WFO (placed as the accepted name of a taxon) |
| 154 | *Impatiens hydrogetonoides* Launert | YES | NO | NO | Zambia | Launert | 1962 | **synonym of *Impatiens* *oreocallis* Hook.f.; accepted name in the genus *Impatiens*** |
| 155 | *Impatiens hypophylla* Makino | YES | NO | NO | Japan | Makino | 1911 | accepted by WFO (placed as the accepted name of a taxon) |
| 156 | *Impatiens hypophylla* Makino | YES | NO | NO | Japan | Utami and Shimizu | 2005 | accepted by WFO (placed as the accepted name of a taxon) |
| 157 | *Impatiens inconspicua* Benth. ex Wight & Arn. | YES | NO | NO | India | Wight and Walker-Arnott | 1834 | accepted by WFO (placed as the accepted name of a taxon) |
| 158 | *Impatiens inconspicua* Benth. ex Wight & Arn. | YES | NO | NO | India | India Biodiversity Portal (https://indiabiodiversity.org/species/show/230016) | 2024 | accepted by WFO (placed as the accepted name of a taxon) |
| 159 | *Impatiens indo-chinensis* Hook.f. | YES | NO | YES | Cambodia | *Hooker | 1908 | unplaced by WFO (a taxonomist hasn’t yet placed the name in the taxonomy), found on IPNI |
| 160 | *Impatiens infundibularis* Hook.f. | NO | YES | NO | Nepal | Raskoti and Ale | 2022 | accepted by WFO (placed as the accepted name of a taxon) |
| 161 | *Impatiens irvingii* Hook.f. | NO | NO | YES | Sudan | Andrews | 1950 | accepted by WFO (placed as the accepted name of a taxon) |
| 162 | *Impatiens jangjeonense* Ami Oh, Byoung-Un Oh & Hyun Kyung Oh | YES | YES | YES | Korea | Oh et al. | 2024 | unplaced by WFO (a taxonomist hasn’t yet placed the name in the taxonomy), found on IPNI |
| 163 | *Impatiens jiulongshanica* Y.L.Xu & Y.L.Chen | YES | NO | YES | China | Xu and Chen | 1999 | accepted by WFO (placed as the accepted name of a taxon) |
| 164 | *Impatiens joachimii* G.M.Schulze | YES | NO | NO | Tanzania | Grey-Wilson | 1980 | accepted by WFO (placed as the accepted name of a taxon) |
| 165 | *Impatiens johnii* E.Barnes | YES | NO | NO | India | India Biodiversity Portal  (https://indiabiodiversity.org/species/show/263000) | 2024 | accepted by WFO (placed as the accepted name of a taxon) |
| 166 | *Impatiens johnsiana* Rateesh, Sunil & Anil Kumar | YES | NO | YES | India | °Narayanan et al. | 2012 | accepted by WFO (placed as the accepted name of a taxon) |
| 167 | *Impatiens josephia* Sinj.Thomas, B.Mani & Britto | YES | YES | NO | India | Mani et al. | 2018 | accepted by WFO (placed as the accepted name of a taxon) |
| 168 | *Impatiens kachinensis* Hook.f. ex Toppin | YES | NO | NO | India | Toppin | 1920 | accepted by WFO (placed as the accepted name of a taxon) |
| 169 | *Impatiens kamerunensis* Warb. | YES | NO | NO | Africa (country? | Grey-Wilson | 1980 | accepted by WFO (placed as the accepted name of a taxon) |
| 170 | *Impatiens kamtilongensis* Toppin | YES | NO | NO | Myanmar | Toppin | 1920 | synonym of ***Impatiens khasiana*** Hook.f.; accepted name in the genus *Impatiens* |
| 171 | *Impatiens kanburiensis* T. Shimizu | YES | NO | NO | Thailand | Shimizu | 1991 | accepted by WFO (placed as the accepted name of a taxon |
| 172 | *Impatiens karuppusamyi* P.S.S.Rich. & V.Ravich. | YES | NO | NO | India | Richard and Ravichandran | 2023 | unplaced by WFO (a taxonomist hasn’t yet placed the name in the taxonomy), found on IPNI |
| 173 | *Impatiens kawttyana* Chhabra & Ramneek | YES | YES | NO | India | Chhabra et al. | 2016 | accepted by WFO (placed as the accepted name of a taxon) |
| 174 | *Impatiens keilii* Gilg | YES | NO | NO | Africa (country?) | Grey-Wilson | 1980 | accepted by WFO (placed as the accepted name of a taxon) |
| 175 | *Impatiens kentrodonta* Gilg | YES | NO | NO | Tanzania | Grey-Wilson | 1980 | accepted by WFO (placed as the accepted name of a taxon) |
| 176 | *Impatiens kerriae* Craib | YES | NO | NO | Thailand | Utami and Shimizu | 2005 | accepted by WFO (placed as the accepted name of a taxon) |
| 177 | *Impatiens kilimanjari* Oliv. | YES | NO | NO | Tanzania | Grey-Wilson | 1980 | accepted by WFO (placed as the accepted name of a taxon) |
| 178 | *Impatiens kleiniformis* Sedgw. | YES | NO | NO | India | Kale and Shinde | 2023 | accepted by WFO (placed as the accepted name of a taxon) |
| 179 | *Impatiens kleiniformis* Sedgw. | YES | NO | NO | India | India Biodiversity Portal  (https://indiabiodiversity.org/species/show/264382) | 2024 | accepted by WFO (placed as the accepted name of a taxon) |
| 180 | *Impatiens kleinii* Wight. & Arn. | YES | NO | NO | India | Utami and Shimizu | 2005 | synonym of ***Impatiens minor var. minor.****;* accepted name in the genus *Impatiens* |
| 181 | *Impatiens klemmeana* Hook.f. | YES | NO | NO | Philippines | ******Hooker | 1909 | accepted by WFO (placed as the accepted name of a taxon) |
| 182 | *Impatiens kodachadriensis* Bhaskar & Sringesh | YES | NO | NO | India | Bhaskar | 2012 | accepted by WFO (placed as the accepted name of a taxon) |
| 183 | *Impatiens kodachadriensis* Bhaskar & Sringesh | YES | NO | NO | India | Indian Biodiversity Portal (https://indiabiodiversity.org/species/show/281334) | 2024 | accepted by WFO (placed as the accepted name of a taxon) |
| 184 | *Impatiens kulamavuensis* Pandur. & V.J.Nair | YES | NO | YES | India | Pandurangan and Nair | 1995 | accepted by WFO (placed as the accepted name of a taxon) |
| 185 | *Impatiens kulamavuensis* Pandur. & V.J.Nair | YES | NO | NO | India | India Biodiversity Portal  (https://indiabiodiversity.org/species/show/263001) | 1995 | accepted by WFO (placed as the accepted name of a taxon) |
| 186 | *Impatiens labordei* Hook.f. | YES | NO | NO | China | ***Hooker | 1908 | accepted by WFO (placed as the accepted name of a taxon) |
| 187 | *Impatiens labordei* Hook.f. | YES | NO | NO | China | Chen et al.  eFloras (Flora of China)  (http://www.efloras.org/florataxon.aspx?flora_id=2&taxon_id=242445528) | 2008 | accepted by WFO (placed as the accepted name of a taxon) |
| 188 | *Impatiens lacei* Hook.f. | YES | NO | YES | Myanmar | ****Hooker | 1910 | accepted by WFO (placed as the accepted name of a taxon) |
| 189 | *Impatiens lanessanii* Hook.f. | YES | NO | YES | Vietnam | *Hooker | 1908 | accepted by WFO (placed as the accepted name of a taxon) |
| 190 | *Impatiens langeana* Hook.f. | YES | NO | YES | India | ****Hooker | 1910 | accepted by WFO (placed as the accepted name of a taxon) |
| 191 | *Impatiens langeana* Hook.f. | YES | NO | YES | India | India Biodiversity Portal  (https://indiabiodiversity.org/species/show/281335) | 1910 | accepted by WFO (placed as the accepted name of a taxon) |
| 192 | *Impatiens latebracteata* Hook.f. | YES | NO | NO | China | **Hooker | 1910 | accepted by WFO (placed as the accepted name of a taxon) |
| 193 | *Impatiens latebracteata* Hook.f. | YES | NO | NO | China | Chen et al.  eFloras (Flora of China)  (http://www.efloras.org/florataxon.aspx?flora_id=2&taxon_id=242445529) | 2008 | accepted by WFO (placed as the accepted name of a taxon) |
| 194 | *Impatiens laticornis* C.E.C.Fisch. | YES | YES | YES | India | Tharani | 2021 | accepted by WFO (placed as the accepted name of a taxon) |
| 195 | *Impatiens laticornis* C.E.C.Fisch. | YES | NO | NO | India | India Biodiversity Portal  (https://indiabiodiversity.org/species/show/253691) | 2024 | accepted by WFO (placed as the accepted name of a taxon) |
| 196 | *Impatiens lawsonii* Hook.f. | YES | NO | NO | India | India Biodiversity Portal  (https://indiabiodiversity.org/species/show/263003) | 2024 | accepted by WFO (placed as the accepted name of a taxon) |
| 197 | *Impatiens lecomtei* Hook.f. | YES | NO | NO | China | ***Hooker | 1908 | accepted by WFO (placed as the accepted name of a taxon) |
| 198 | *Impatiens lecomtei* Hook.f. | YES | NO | NO | China | Chen et al.  eFloras (Flora of China)  (http://www.efloras.org/florataxon.aspx?flora_id=2&taxon_id=242445530) | 2008 | accepted by WFO (placed as the accepted name of a taxon) |
| 199 | *Impatiens leedalii* Grey-Wilson | YES | NO | NO | Tanzania | Grey-Wilson | 1980 | accepted by WFO (placed as the accepted name of a taxon) |
| 200 | *Impatiens lenta* Hook.f. | YES | NO | YES | India | ****Hooker | 1910 | accepted by WFO (placed as the accepted name of a taxon) |
| 201 | *Impatiens lenta* Hook.f. | YES | NO | NO | India | India Biodiversity Portal  (https://indiabiodiversity.org/species/show/264384) | 2024 | accepted by WFO (placed as the accepted name of a taxon) |
| 202 | *Impatiens leptura* Hook.f. | YES | NO | NO | India | ?Hooker | 1875-1879 | accepted by WFO (placed as the accepted name of a taxon) |
| 203 | *Impatiens leptura* Hook.f. | YES | NO | NO | India | India Biodiversity Portal  (https://indiabiodiversity.org/species/show/263004) | 2024 | accepted by WFO (placed as the accepted name of a taxon) |
| 204 | *Impatiens letestuana* N.Hallé | YES | NO | YES | Gabon | Hallé | 1962 | accepted by WFO (placed as the accepted name of a taxon) |
| 205 | *Impatiens letestuana* N.Hallé | YES | NO | NO | Gabon | Grey-Wilson | 1980 | accepted by WFO (placed as the accepted name of a taxon) |
| 206 | *Impatiens leveillei* Hook.f. | YES | NO | YES | China | *Hooker | 1908 | accepted by WFO (placed as the accepted name of a taxon) |
| 207 | *Impatiens leveillei* Hook.f. | YES | NO | NO | China | Chen et al.  eFloras  (Flora of China)  (http://www.efloras.org/florataxon.aspx?flora_id=2&taxon_id=242445533) | 2008 | accepted by WFO (placed as the accepted name of a taxon) |
| 208 | *Impatiens liangshanensis* Q.Luo | YES | NO | NO | China | Luo | 2011 | accepted by WFO (placed as the accepted name of a taxon) |
| 209 | *Impatiens ligulata* Bedd. | YES | NO | NO | India | Beddome | 1859 | accepted by WFO (placed as the accepted name of a taxon) |
| 210 | *Impatiens lilacina* Hook.f. | YES | NO | NO | China | ***Hooker | 1908 | accepted by WFO (placed as the accepted name of a taxon) |
| 211 | *Impatiens lilacina* Hook.f. | YES | NO | NO | China | Chen et al.  eFloras  (Flora of China) (http://www.efloras.org/florataxon.aspx?flora_id=2&taxon_id=242445534) | 2008 | accepted by WFO (placed as the accepted name of a taxon) |
| 212 | *Impatiens limnophila* Launert | YES | NO | NO | Zambia | Launert | 1962 | accepted by WFO (placed as the accepted name of a taxon) |
| 213 | *Impatiens lohitensis* Gogoi & Borah | YES | NO | NO | India | Gogoi and Borah | 2013 | accepted by WFO (placed as the accepted name of a taxon) |
| 214 | *Impatiens longecalcarata* Tardieu | YES | NO | NO | Laos | Tardieu | 1944 | accepted by WFO (placed as the accepted name of a taxon) |
| 215 | *Impatiens longirama* Hook.f. | YES | NO | NO | India | ^Hooker | 1910 | accepted by WFO (placed as the accepted name of a taxon) |
| 216 | *Impatiens longirama* Hook.f. | YES | NO | NO | India | India Biodiversity Portal  (https://indiabiodiversity.org/species/show/281340) | 2024 | accepted by WFO (placed as the accepted name of a taxon) |
| 217 | *Impatiens lukwangulensis* Grey-Wilson | YES | NO | NO | Tanzania | Grey-Wilson | 1980 | accepted by WFO (placed as the accepted name of a taxon) |
| 218 | *Impatiens mackeyana* Hook.f. | YES | NO | NO | Africa (Cameroon) | ********Hooker | 1863 | accepted by WFO (placed as the accepted name of a taxon) |
| 219 | *Impatiens mackeyana* Hook.f. | YES | NO | NO | Africa (country?) | Grey-Wilson | 1980 | accepted by WFO (placed as the accepted name of a taxon) |
| 220 | *Impatiens macrocarpa* Hook.f. | YES | NO | NO | India | *******Hooker | 1911 | accepted by WFO (placed as the accepted name of a taxon) |
| 221 | *Impatiens macrocarpa* Hook.f. | YES | NO | NO | India | India Biodiversity Portal  (https://indiabiodiversity.org/species/show/263005) | 2024 | accepted by WFO (placed as the accepted name of a taxon) |
| 222 | *Impatiens macroptera* Hook.f. | YES | NO | NO | Africa (country?) | Grey-Wilson | 1980 | accepted by WFO (placed as the accepted name of a taxon) |
| 223 | *Impatiens macrosepala* Hook.f. | YES | NO | NO | Malaysia | }Hooker | 1909 | accepted by WFO (placed as the accepted name of a taxon) |
| 224 | *Impatiens macrosepala* Hook.f. | YES | NO | NO | Thailand | Utami and Shimizu | 2005 | accepted by WFO (placed as the accepted name of a taxon) |
| 225 | *Impatiens macrovexilla* Y.L.Chen | YES | NO | YES | China | Chen | 2000 | accepted by WFO (placed as the accepted name of a taxon) |
| 226 | *Impatiens macrovexilla* Y.L.Chen | YES | NO | YES | China | Chen et al.  eFloras (Flora of China)  (http://www.efloras.org/florataxon.aspx?flora_id=2&taxon_id=242445539) | 2008 | accepted by WFO (placed as the accepted name of a taxon) |
| 227 | *Impatiens mankulamensis* K.M.P.Kumar, R.Jagad. & Nagaraj | YES | NO | NO | India | Prabhukumar et al. | 2017 | accepted by WFO (placed as the accepted name of a taxon) |
| 228 | *Impatiens mannii* Hook.f. | YES | NO | NO | Africa (country?) | Grey-Wilson | 1980 | accepted by WFO (placed as the accepted name of a taxon) |
| 229 | *Impatiens marivorahonensis* Humbert | YES | NO | NO | Madagascar | Humbert | 1956 | accepted by WFO (placed as the accepted name of a taxon) |
| 230 | *Impatiens masisiensis* De Wild. | YES | NO | NO | Zaire | Grey-Wilson | 1980 | accepted by WFO (placed as the accepted name of a taxon) |
| 231 | *Impatiens masoni* Hook.f. | YES | NO | NO | Thailand | Utami and Shimizu | 2005 | accepted by WFO (placed as the accepted name of a taxon) |
| 232 | *Impatiens matthewiana* Ramas. & Pandur. | YES | YES | YES | India | Ramassubu et al. | 2015 | accepted by WFO (placed as the accepted name of a taxon) |
| 233 | *Impatiens meeboldii* Hook.f. | YES | NO | NO | India | ^Hooker | 1910 | accepted by WFO (placed as the accepted name of a taxon) |
| 234 | *Impatiens meeboldii* Hook.f. | YES | NO | NO | India | India Biodiversity Portal  (https://indiabiodiversity.org/species/show/281348) | 2024 | accepted by WFO (placed as the accepted name of a taxon) |
| 235 | *Impatiens megamalayana* Ramasubbu | YES | YES | YES | India | Ramasubbu et al. | 2017 | accepted by WFO (placed as the accepted name of a taxon) |
| 236 | *Impatiens membranifolia* Franch. ex Hook.f. | YES | NO | NO | China | ***Hooker | 1908 | accepted by WFO (placed as the accepted name of a taxon) |
| 237 | *Impatiens membranifolia* Franch. ex Hook.f. | YES | NO | NO | China | Chen et al.  eFloras (Flora of China)  http://www.efloras.org/florataxon.aspx?flora_id=2&taxon_id=242445543 | 2008 | accepted by WFO (placed as the accepted name of a taxon) |
| 238 | *Impatiens mexicana* Rydb | YES | YES | NO | México | Monzalvo et al. | 2024 | accepted by WFO (placed as the accepted name of a taxon) |
| 239 | *Impatiens microstachys* Hook.f. | YES | NO | NO | China | **Hooker | 1910 | accepted by WFO (placed as the accepted name of a taxon) |
| 240 | *Impatiens microstachys* Hook.f. | YES | NO | NO | China | Chen et al.  eFloras (Flora of China)  (http://www.efloras.org/florataxon.aspx?flora_id=2&taxon_id=242445547) | 2008 | accepted by WFO (placed as the accepted name of a taxon) |
| 241 | *Impatiens microtheca* Hook. f. | YES | NO | YES | India | ****Hooker | 1910 | synonym of ***Impatiens goughii*** Wight.; accepted name in the genus *Impatiens* |
| 242 | *Impatiens mildbraedii* Gilg | YES | NO | NO | Africa (country?) | Grey-Wilson | 1980 | accepted by WFO (placed as the accepted name of a taxon) |
| 243 | *Impatiens minor* (DC.) Bennet | YES | NO | NO | India | India Biodiversity Portal  (https://indiabiodiversity.org/species/show/230018) | 2024 | accepted by WFO (placed as the accepted name of a taxon) |
| 244 | *Impatiens modesta* Wight | YES | NO | NO | India | India Biodiversity Portal  (https://indiabiodiversity.org/species/show/253706) | 2024 | accepted by WFO (placed as the accepted name of a taxon) |
| 245 | *Impatiens mohana* Ratheesh, Sujana & Anil Kumar | YES | NO | NO | India | Narayanan et al. | 2012 | accepted by WFO (placed as the accepted name of a taxon) |
| 246 | *Impatiens monotricha* Hook.f. | YES | NO | YES | Laos | ****Hooker | 1910 | unplaced by WFO (a taxonomist hasn’t yet placed the name in the taxonomy) found on IPNI |
| 247 | *Impatiens mullaingiriensis* Bhaskar | YES | NO | NO | India | India Biodiversity Portal  (https://indiabiodiversity.org/species/show/281351) | 2024 | accepted by WFO (placed as the accepted name of a taxon) |
| 248 | *Impatiens munronii* Wight | YES | NO | NO | India | India Biodiversity Flora  (https://indiabiodiversity.org/species/show/253696) | 2024 | accepted by WFO (placed as the accepted name of a taxon) |
| 249 | *Impatiens mussoti* Hook.f. | YES | NO | NO | China | ***Hooker | 1908 | accepted by WFO (placed as the accepted name of a taxon) |
| 250 | *Impatiens mysorensis* Roth | YES | NO | NO | India | India Biodiversity Portal  (https://indiabiodiversity.org/species/show/253690) | 2024 | accepted by WFO (placed as the accepted name of a taxon) |
| 251 | *Impatiens nalamponii* Shimizu | YES | NO | NO | Thailand | Shimizu | 1969 | accepted by WFO (placed as the accepted name of a taxon) |
| 252 | *Impatiens nana* Engl. & Warb. ex Engl. | YES | NO | NO | Africa (country?) | Grey-Wilson | 1980 | accepted by WFO (placed as the accepted name of a taxon) |
| 253 | *Impatiens nayongensis* Chen, He, Li, Huang and Bai | YES | YES | NO | China | Chen et al. | 2022 | not found on WFO or IPNI |
| 254 | *Impatiens neglecta* Y.L.Xu & Y.L.Chen | YES | NO | YES | China | Xu and Chen | 1999 | accepted by WFO (placed as the accepted name of a taxon) |
| 255 | *Impatiens neglecta* Y.L.Xu & Y.L.Chen | YES | NO | YES | China | Chen et al.  eFlora of China  (http://www.efloras.org/florataxon.aspx?flora_id=2&taxon_id=242326703) | 2008 | accepted by WFO (placed as the accepted name of a taxon) |
| 256 | *Impatiens neobarnesii* C.E.C.Fisch. | YES | NO | NO | India | India Biodiversity Portal  (https://indiabiodiversity.org/species/show/253677) | 2024 | accepted by WFO (placed as the accepted name of a taxon) |
| 257 | *Impatiens neo-modesta* Hareesh, K.M.P.Kumar & V.B.Sreek. | YES | YES | YES | India | Prabhukumar et al. | 2015 | accepted by WFO (placed as the accepted name of a taxon) |
| 258 | *Impatiens neomunronii* L.Joseph & Bhaskar | YES | NO | NO | India | India Biodiversity Portal  (https://indiabiodiversity.org/species/show/281352) | 2024 | accepted by WFO (placed as the accepted name of a taxon) |
| 259 | *Impatiens nepalensis* Hook.f. | YES | NO | NO | Nepal | *****Hooker | 1911 | synonym of ***Impatiens bicolor*** Royle.; accepted name in the genus *Impatiens* |
| 260 | *Impatiens niamniamensis* Gilg | YES | NO | NO | Africa (country)) | Grey-Wilson | 1980 | accepted by WFO (placed as the accepted name of a taxon) |
| 261 | *Impatiens nigrescens* Hook.f. | YES | NO | NO | India | India Biodiversity Portal  (https://indiabiodiversity.org/species/show/281353) | 2024 | accepted by WFO (placed as the accepted name of a taxon) |
| 262 | *Impatiens nilgirica* C.E.C.Fisch. | YES | NO | NO | India | India Biodiversity Portal  (https://indiabiodiversity.org/species/show/253754) | 2024 | accepted by WFO (placed as the accepted name of a taxon) |
| 263 | *Impatiens nimspurjae* Raskoti | YES | YES | YES | Nepal | Raskoti and Ale | 2022 | accepted by WFO (placed as the accepted name of a taxon) |
| 264 | *Impatiens nobilis* Hook.f. | YES | NO | NO | China | ***Hooker | 1908 | accepted by WFO (placed as the accepted name of a taxon) |
| 265 | *Impatiens nobilis* Hook.f. | YES | NO | NO | China | Chen et al.  eFlora (Flora of China)  (http://www.efloras.org/florataxon.aspx?flora_id=2&taxon_id=242445555) | 2008 | accepted by WFO (placed as the accepted name of a taxon) |
| 266 | *Impatiens notolopha* Maxim. | YES | NO | NO | China | *****Hooker | 1911 | accepted by WFO (placed as the accepted name of a taxon) |
| 267 | *Impatiens notolopha* Maxim. | YES | NO | NO | China | Chen et al.  eFlora (Flora of China)  (http://www.efloras.org/florataxon.aspx?flora_id=2&taxon_id=242445556) | 2008 | accepted by WFO (placed as the accepted name of a taxon) |
| 268 | *Impatiens notoptera* Hook.f. | YES | NO | NO | Cambodia | #Hooker | 1909 | accepted by WFO (placed as the accepted name of a taxon) |
| 269 | *Impatiens notoptera* Hook.f. | YES | NO | YES | Cambodia | ****Hooker | 1910 | accepted by WFO (placed as the accepted name of a taxon) |
| 270 | *Impatiens nubigena* W.W.SM. | YES | NO | NO | China | Smith | 1914 | accepted by WFO (placed as the accepted name of a taxon) |
| 271 | *Impatiens nubigena* W.W.SM. | YES | NO | NO | China | Chen et al.  eFlora  (Flora of China)  (http://www.efloras.org/florataxon.aspx?flora_id=2&taxon_id=242326704) | 2008 | accepted by WFO (placed as the accepted name of a taxon) |
| 272 | *Impatiens nzoana* A.Chev. | YES | NO | NO | Africa (country) | Grey-Wilson | 1980 | accepted by WFO (placed as the accepted name of a taxon) |
| 273 | *Impatiens obscura* Hook.f. | YES | NO | NO | Vietnam | #Hooker | 1909 | accepted by WFO (placed as the accepted name of a taxon) |
| 274 | *Impatiens oncidioides* Ridl. Ex Hook.f. | YES | NO | NO | Malaysia | }Hooker | 1909 | accepted by WFO (placed as the accepted name of a taxon) |
| 275 | *Impatiens oppositifolia* L. | YES | NO | NO | India | India Biodiversity Portal  (https://indiabiodiversity.org/species/show/230019) | 2024 | accepted by WFO (placed as the accepted name of a taxon) |
| 276 | *Impatiens orchioides* Bedd. | YES | NO | NO | India | Beddome | 1864 | accepted by WFO (placed as the accepted name of a taxon) |
| 277 | *Impatiens orchioides* Bedd. | YES | NO | NO | India | India Biodiversity Portal  (https://indiabiodiversity.org/species/show/263007) | 2024 | accepted by WFO (placed as the accepted name of a taxon) |
| 278 | *Impatiens oreocallis* Launert | YES | NO | NO | Malawi | Launert | 1962 | accepted by WFO (placed as the accepted name of a taxon) |
| 279 | *Impatiens oreocallis* Launert | YES | NO | NO | Africa (country?) | Grey-Wilson | 1980 | accepted by WFO (placed as the accepted name of a taxon) |
| 280 | *Impatiens palpebrata* Hook.f. | YES | NO | YES | Gabon | Hallé | 1962 | accepted by WFO (placed as the accepted name of a taxon) |
| 281 | *Impatiens palpebrata* Hook.f. | YES | NO | NO | Africa (country?) | Grey-Wilson | 1980 | accepted by WFO (placed as the accepted name of a taxon) |
| 282 | *Impatiens panduranganii* K.M.P.Kumar, R.Jagad. & G.Prasad | YES | YES | NO | India | Prabhukumar | 2017 | accepted by WFO (placed as the accepted name of a taxon) |
| 283 | *Impatiens paradoxa* C.S.Zhu & H.W.Yang | YES | NO | NO | China | Zhu and Yang | 1994 | accepted by WFO (placed as the accepted name of a taxon) |
| 284 | *Impatiens paradoxa* C.S.Zhu & H.W.Yang | YES | NO | NO | China | Chen et al.  eFloras  (Flora of China)  (http://www.efloras.org/florataxon.aspx?flora_id=2&taxon_id=242326706) | 1994 | accepted by WFO (placed as the accepted name of a taxon) |
| 285 | *Impatiens pathakiana* Gogoi & Borah | YES | NO | NO | India | Gogoi and Borah | 2015 | accepted by WFO (placed as the accepted name of a taxon) |
| 286 | *Impatiens pahalgamensis* Hook.f. | YES | NO | NO | India | *******Hooker | 1911 | accepted by WFO (placed as the accepted name of a taxon) |
| 287 | *Impatiens pahalgamensis* Hook.f. | YES | NO | NO | India | India Biodiversity Portal  (https://indiabiodiversity.org/species/show/281357) | 2024 | accepted by WFO (placed as the accepted name of a taxon) |
| 288 | *Impatiens paludosa* Hook.f. | YES | NO | NO | India | ?Hooker | 1875 | accepted by WFO (placed as the accepted name of a taxon) |
| 289 | *Impatiens pandata* E.Barnes | YES | NO | NO | India | India Biodiversity Portal  (https://indiabiodiversity.org/species/show/253688) | 2024 | accepted by WFO (placed as the accepted name of a taxon) |
| 290 | *Impatiens parasitica* Bedd. | YES | NO | NO | India | Beddome | 1859 | accepted by WFO (placed as the accepted name of a taxon) |
| 291 | *Impatiens parasitica* Bedd. | YES | NO | NO | India | India Biodiversity Portal  (https://indiabiodiversity.org/species/show/263009) | 2024 | accepted by WFO (placed as the accepted name of a taxon) |
| 292 | *Impatiens pellegrini* N. Halle | YES | NO | YES | Gabon | Hallé | 1962 | synonym of ***Impatiens gossweileri subsp. gossweileri***.; accepted name in the genus *Impatiens* |
| 293 | *Impatiens palliderosea* Gilg | YES | NO | NO | Tanzania | Grey-Wilson | 1980 | accepted by WFO (placed as the accepted name of a taxon) |
| 294 | *Impatiens pendula* B.Heyne ex Wight & Arn. | YES | NO | NO | India | India Biodiversity Portal  https://indiabiodiversity.org/species/show/253756 | 2024 | accepted by WFO (placed as the accepted name of a taxon) |
| 295 | *Impatiens periyarensis* B. Mani, Sinj. Thomas & Britto | YES | YES | NO | India | Mani et al. | 2020 | accepted by WFO (placed as the accepted name of a taxon) |
| 296 | *Impatiens perrottetii* Turcz. | YES | NO | YES | India | ****Hooker | 1910 | synonym of ***Impatiens inconspicua var. inconspicua****.*; accepted name in the genus *Impatiens* |
| 297 | *Impatiens phoenicea* Bedd. | YES | NO | NO | India | India Biodiversity Portal  https://indiabiodiversity.org/species/show/263010 | 2024 | accepted by WFO (placed as the accepted name of a taxon) |
| 298 | *Impatiens phuluangensis* Shimizu | YES | NO | NO | Thailand | Shimizu | 1969 | accepted by WFO (placed as the accepted name of a taxon) |
| 299 | *Impatiens platyadena* C.E.C.Fisch. | YES | NO | NO | India | Fischer | 1934 | accepted by WFO (placed as the accepted name of a taxon) |
| 300 | *Impatiens platyadena* C.E.C.Fisch. | YES | NO | NO | India | India Biodiversity Portal  (https://indiabiodiversity.org/species/show/253733) | 2024 | accepted by WFO (placed as the accepted name of a taxon) |
| 301 | *Impatiens podocarpa* Hook.f. | YES | NO | YES | India | *****Hooker | 1911 | accepted by WFO (placed as the accepted name of a taxon) |
| 302 | *Impatiens podocarpa* Hook.f. | YES | NO | NO | India | India Biodiversity Portal  (https://indiabiodiversity.org/species/show/281360) | 2024 | accepted by WFO (placed as the accepted name of a taxon) |
| 303 | *Impatiens polyantha* Gilg | YES | NO | NO | Africa (country?) | Grey-Wilson | 1980 | accepted by WFO (placed as the accepted name of a taxon) |
| 304 | *Impatiens polyceras* Hook.f. ex W.W.Sm. | YES | NO | NO | China | Smith | 1915 | accepted by WFO (placed as the accepted name of a taxon) |
| 305 | *Impatiens polyceras* Hook.f. ex W.W.Sm. | YES | NO | NO | China | Chen et al.  eFloras (Flora of China)  (http://www.efloras.org/florataxon.aspx?flora_id=2&taxon_id=242326709) | 2008 | accepted by WFO (placed as the accepted name of a taxon) |
| 306 | *Impatiens porrecta* Wall. | YES | NO | NO | Burma | Toppin | 1920 | accepted by WFO (placed as the accepted name of a taxon) |
| 307 | *Impatiens porrecta* Wall. | YES | NO | NO | India | India Biodiversity Flora (https://indiabiodiversity.org/species/show/253711) | 2024 | accepted by WFO (placed as the accepted name of a taxon) |
| 308 | *Impatiens potaninii* Maxim. | YES | NO | NO | China | *****Hooker | 1911 | accepted by WFO (placed as the accepted name of a taxon) |
| 309 | *Impatiens prainii* Hook.f. | YES | NO | YES | India | *****Hooker | 1911 | unplaced by WFO (a taxonomist hasn’t yet placed the name in the taxonomy), found on IPNI |
| 310 | *Impatiens prainii* Hook.f. | YES | NO | NO | India | India Biodiversity Portal  https://indiabiodiversity.org/species/show/281362 | 1911 | unplaced by WFO (a taxonomist hasn’t yet placed the name in the taxonomy), found on IPNI |
| 311 | *Impatiens preussii* Warb. | YES | NO | NO | Cameroon | Warburg | 1897 | accepted by WFO (placed as the accepted name of a taxon) |
| 312 | *Impatiens pritchardii* Toppin | YES | NO | NO | Myanmar | Toppin | 1920 | synonym of ***Impatiens khasiana*** Hook.f.; accepted name in the genus *Impatiens* |
| 313 | *Impatiens procumbens* Franch. | YES | NO | NO | China | ***Hooker | 1908 | accepted by WFO (placed as the accepted name of a taxon) |
| 314 | *Impatiens proctrata* Hook.f. | YES | NO | NO | Vietnam | #Hooker | 1909 | accepted by WFO (placed as the accepted name of a taxon); |
| 315 | *Impatiens proctrata* Hook.f. | YES | NO | NO | Indochina | Utami and Shimizu | 2005 | accepted by WFO (placed as the accepted name of a taxon); |
| 316 | *Impatiens prostrata* Hook.f. | YES | NO | NO | India | India Biodiversity Portal  (https://indiabiodiversity.org/species/show/281363) | 2024 | accepted by WFO (placed as the accepted name of a taxon) |
| 317 | *Impatiens pseudoacaulis* Bhaskar | YES | NO | NO | India | Bhaskar | 2012 | accepted by WFO (placed as the accepted name of a taxon) |
| 318 | *Impatiens pseudoacaulis* Bhaskar | YES | NO | NO | India | India Biodiversity Portal (https://indiabiodiversity.org/species/show/281364) | 2024 | accepted by WFO (placed as the accepted name of a taxon) |
| 319 | *Impatiens pseudokingii* Hand.-Mazz. | YES | NO | NO | China | Handel-Mazzetti | 1933 | accepted by WFO (placed as the accepted name of a taxon) |
| 320 | *Impatiens pseudokingii* Hand.-Mazz. | YES | NO | NO | China | Chen et al.  eFloras (Flora of China)  (http://www.efloras.org/florataxon.aspx?flora_id=2&taxon_id=242445571) | 2008 | accepted by WFO (placed as the accepted name of a taxon) |
| 321 | *Impatiens pseudomacroptera* Grey-Wilson | YES | NO | NO | Gabon | Grey-Wilson | 1980 | accepted by WFO (placed as the accepted name of a taxon) |
| 322 | *Impatiens psychadelpoides* Launert | YES | NO | NO | Mozambique | Launert | 1962 | accepted by WFO (placed as the accepted name of a taxon) |
| 323 | *Impatiens psychadelphoides* Launert | YES | NO | NO | Mozambique | Grey-Wilson | 1980 | accepted by WFO (placed as the accepted name of a taxon) |
| 324 | *Impatiens pulcherrima* Dalzell | YES | NO | NO | India | Dalzell | 1850 | accepted by WFO (placed as the accepted name of a taxon) |
| 325 | *Impatiens pulcherrima* Dalzell | YES | NO | NO | India | India Biodiversity Portal  (https://indiabiodiversity.org/species/show/253686) | 2024 | accepted by WFO (placed as the accepted name of a taxon) |
| 326 | *Impatiens pulchra* Hook. f. & Thomson | YES | NO | NO | India | Hooker and Thomson | 1859 | accepted by WFO (placed as the accepted name of a taxon) |
| 327 | *Impatiens pulchra* Hook. f. & Thomson | YES | NO | NO | India | Rewicz et al. | 2020 | accepted by WFO (placed as the accepted name of a taxon) |
| 328 | *Impatiens pulchra* Hook. f. & Thomson | YES | NO | NO | India | India Biodiversity Portal  (https://indiabiodiversity.org/species/show/253685) | 2020 | accepted by WFO (placed as the accepted name of a taxon) |
| 329 | *Impatiens quingchengshanica* Y.M.Yuan, Y.Song & X.J.Ge | YES | NO | NO | China | Yuan et al. | 2011 | accepted by WFO (placed as the accepted name of a taxon) |
| 330 | *Impatiens quisqualis* Launert | YES | NO | NO | Malawi | Launert | 1962 | accepted by WFO (placed as the accepted name of a taxon)  203 |
| 331 | *Impatiens raphidothrix* Warb. | YES | NO | NO | Africa (country) | Grey-Wilson | 1980 | accepted by WFO (placed as the accepted name of a taxon)  203 |
| 332 | *Impatiens raziana* Bashkar & Razi | YES | NO | NO | India | Bhaskar and Razi | 1982 | accepted by WFO (placed as the accepted name of a taxon) |
| 333 | *Impatiens raziana* Bashkar & Razi | YES | NO | NO | India | India Biodiversity Portal  (https://indiabiodiversity.org/species/show/281367) | 2024 | accepted by WFO (placed as the accepted name of a taxon) |
| 334 | *Impatiens recurvicornis* Maxim. | YES | NO | NO | China | Chen et al.  eFloras (Flora of China)  (http://www.efloras.org/florataxon.aspx?flora_id=2&taxon_id=242445575) | 2008 | accepted by WFO (placed as the accepted name of a taxon) |
| 335 | *Impatiens reidii* Hook.f. | YES | NO | YES | India | ****Hooker | 1910 | accepted by WFO (placed as the accepted name of a taxon) |
| 336 | *Impatiens reidii* Hook.f. | YES | NO | NO | India | India Biodiversity  (https://indiabiodiversity.org/species/show/281368) | 2024 | accepted by WFO (placed as the accepted name of a taxon) |
| 337 | *Impatiens ridleyi* Hook.f. | YES | NO | NO | Malaysia | }Hooker | 1909 | accepted by WFO (placed as the accepted name of a taxon) |
| 338 | *Impatiens rivulicola* Hook.f. | YES | NO | NO | India | ******* Hooker | 1911 | accepted by WFO (placed as the accepted name of a taxon) |
| 339 | *Impatiens rosulata* Grey-Wilson | YES | NO | NO | Tanzania | Grey-Wilson | 1980 | accepted by WFO (placed as the accepted name of a taxon) |
| 340 | *Impatiens rothii* Hook.f. | YES | NO | NO | Ethiopia | Grey-Wilson | 1980 | accepted by WFO (placed as the accepted name of a taxon) |
| 341 | *Impatiens rubrolineata* Hook.f. | YES | NO | NO | Myanmar | Toppin | 1920 | synonym of ***Impatiens laevigata var. laevigata.****;* accepted name in the genus *Impatiens* |
| 342 | *Impatiens rubromaculata* Warb. | YES | NO | NO | Africa (country?) | Grey-Wilson | 1980 | accepted by WFO (placed as the accepted name of a taxon) |
| 343 | *Impatiens rupicola* Hook.f. | YES | NO | NO | India | ^Hooker | 1910 | accepted by WFO (placed as the accepted name of a taxon) |
| 344 | *Impatiens rupicola* Hook.f. | YES | NO | NO | India | India Biodiversity Portal  (https://indiabiodiversity.org/species/show/264386) | 2024 | accepted by WFO (placed as the accepted name of a taxon) |
| 345 | *Impatiens ruthiae* Suksathan & Triboun | YES | NO | NO | Thailand | Suksathan and Triboun | 2009 | accepted by WFO (placed as the accepted name of a taxon) |
| 346 | *Impatiens sahyadrica* V.B. Sreek., Hareesh, Dantas & Sujanapal | YES | YES | YES | India | Hareesh et al. | 2015 | accepted by WFO (placed as the accepted name of a taxon) |
| 347 | *Impatiens sakeriana* Hook.f. | YES | NO | NO | Africa (country?) | Grey-Wilson | 1980 | accepted by WFO (placed as the accepted name of a taxon) |
| 348 | *Impatiens salaengensis* T.Shimizu | YES | NO | YES | Thailand | Shimizu | 1991 | accepted by WFO (placed as the accepted name of a taxon) |
| 349 | *Impatiens salicifolia* Baill. | NO | NO | YES | Madagascar | Baillon | 1891 | synonym of ***Impatiens rutenbergii*** Hook.f.; accepted name in the genus *Impatiens* |
| 350 | *Impatiens saliensis* G.M.Schulze | YES | NO | NO | Tanzania | Grey-Wilson | 1980 | accepted by WFO (placed as the accepted name of a taxon) |
| 351 | *Impatiens salpinx* G.M. Schulze & Launert | YES | NO | NO | Zimbabwe and  Mozambique | Launert | 1962 | accepted by WFO (placed as the accepted name of a taxon) |
| 352 | *Impatiens santisukii* T.Shimizu | YES | NO | YES | Thailand | Shimizu | 2000 | accepted by WFO (placed as the accepted name of a taxon) |
| 353 | *Impatiens saraburiensis* T. Shimizu | YES | NO | NO | Thailand | Utami and Shimizu | 2005 | not found on WFO or IPNI |
| 354 | *Impatiens sasidharanii* K.M.P.Kumar, Omalsree, Hareesh & V.B. Sreek. | YES | YES | YES | India | ¨Prabhukumar et al. | 2015 | accepted by WFO (placed as the accepted name of a taxon) |
| 355 | *Impatiens sasidharanii var. hirsuta* K.M.P.Kumar, Omalsree, Hareesh & V.B. Sreek. | YES | YES | NO | India | ¨Prabhukumar et al. | 2015 | accepted by WFO (placed as the accepted name of a taxon) |
| 356 | *Impatiens saulierea* B. Mani, S. Thomas & Britto | YES | YES | NO | India | Mani et al. | 2018 | accepted by WFO (placed as the accepted name of a taxon) |
| 357 | *Impatiens scabriuscula* (G.Don) B.Heyne ex Wall. | YES | NO | NO | India | India Biodiversity Portal  (https://indiabiodiversity.org/species/show/253678) | 2024 | accepted by WFO (placed as the accepted name of a taxon) |
| 358 | *Impatiens scapiflora* Heyne | YES | NO | NO | India | Utami and Shimizu | 2005 | synonym of ***Impatiens acaulis* var. *acaulis***.; accepted name in the genus *Impatiens* |
| 359 | *Impatiens scapiflora* Heyne | YES | NO | NO | India | India Biodiversity Portal  (https://indiabiodiversity.org/species/show/281371) | 2024 | accepted by WFO (placed as the accepted name of a taxon) |
| 360 | *Impatiens shevaroyensis* Bhaskar | YES | NO | NO | India | India Biodiversity Flora  https://indiabiodiversity.org/species/show/281375 | 2024 | accepted by WFO (placed as the accepted name of a taxon) |
| 361 | *Impatiens shirensis* Baker f. | YES | NO | NO | Malawi | Grey-Wilson | 1980 | accepted by WFO (placed as the accepted name of a taxon) |
| 362 | *Impatiens sholayarensis* M.Kumar & Sequiera | YES | NO | YES | India | Kumar and Sequiera | 2001 | accepted by WFO (placed as the accepted name of a taxon) |
| 363 | *Impatiens sholayarensis* M.Kumar & Sequiera | YES | NO | YES | India | India Biodiversity Portal  (https://indiabiodiversity.org/species/show/263011) | 2024 | accepted by WFO (placed as the accepted name of a taxon) |
| 364 | *Impatiens schulziana* Launert | YES | NO | NO | Malawi | Launert | 1962 | synonym of ***Impatiens rubromaculata subsp. schulziana*** (Launert) Grey-Wilson.; accepted name in the genus *Impatiens* |
| 365 | *Impatiens scitula* Hook.f. | YES | NO | NO | India | India Biodiversity Portal  (https://indiabiodiversity.org/species/show/281372) | 2024 | accepted by WFO (placed as the accepted name of a taxon) |
| 366 | *Impatiens scortechinii* Hook.f. | YES | NO | NO | Malaysia | }Hooker | 1909 | accepted by WFO (placed as the accepted name of a taxon) |
| 367 | *Impatiens scutisepala* Hook.f. | YES | NO | YES | China | *****Hooker | 1911 | accepted by WFO (placed as the accepted name of a taxon) |
| 368 | *Impatiens scutisepala* Hook.f. | YES | NO | NO | China | Chen et al.  eFloras (Flora of China)  (http://www.efloras.org/florataxon.aspx?flora_id=2&taxon_id=242445581) | 2008 | accepted by WFO (placed as the accepted name of a taxon) |
| 369 | *Impatiens semounensis* Hook.f. | YES | NO | YES | Indo-China | *Hooker | 1908 | unplaced by WFO (a taxonomist hasn’t yet placed the name in the taxonomy), found on IPNI |
| 370 | *Impatiens serratifolia* Hook.f. | NO | YES | NO | Nepal | Raskoti and Ale | 2022 | accepted by WFO (placed as the accepted name of a taxon) |
| 371 | *Impatiens serratifolia* Hook.f. | YES | NO | NO | Nepal | India Biodiversity Portal  (https://indiabiodiversity.org/species/show/281374) | 2024 | accepted by WFO (placed as the accepted name of a taxon) |
| 372 | *Impatiens sigmoidea* Hook.f. | YES | NO | NO | China | ***Hooker | 1908 | accepted by WFO (placed as the accepted name of a taxon) |
| 373 | *Impatiens sigmoidea* Hook.f. | YES | NO | NO | China | Chen et al.  eFloras  (Flora of China)  (http://www.efloras.org/florataxon.aspx?flora_id=2&taxon_id=242445582) | 2008 | accepted by WFO (placed as the accepted name of a taxon) |
| 374 | *Impatiens sikkimensis* Govaerts & Chakrab. | YES | YES | NO | India | Gogoi et al. | 2018 | synonym of ***Impatiens humilis*** Hook.f.; accepted name in the genus *Impatiens* |
| 375 | *Impatiens smitinandii* Shimizu | YES | YES | NO | Thailand | Shimizu | 1969 | accepted by WFO (placed as the accepted name of a taxon) |
| 376 | *Impatiens spissiflora* Hook.f. | YES | NO | NO | India | ^Hooker | 1910 | accepted by WFO (placed as the accepted name of a taxon) |
| 376 | *Impatiens spissiflora* Hook.f. | YES | NO | NO | India | India Biodiversity Portal  (https://indiabiodiversity.org/species/show/281377) | 1910 | accepted by WFO (placed as the accepted name of a taxon) |
| 377 | *Impatiens sodenii* Engl. & Warb. ex Engl. | YES | NO | NO | Africa (Country?) | Grey-Wilson | 1980 | accepted by WFO (placed as the accepted name of a taxon) |
| 378 | *Impatiens spectabilis* Triboun & Suksathan | YES | NO | NO | Thailand | Suksathan and Triboun | 2009 | accepted by WFO (placed as the accepted name of a taxon) |
| 379 | *Impatiens stoliczkai* Hook.f. | YES | NO | YES | India | ****Hooker | 1910 | accepted by WFO (placed as the accepted name of a taxon) |
| 380 | *Impatiens stoliczkai* Hook.f. | YES | NO | YES | India | India Biodiversity Flora  (https://indiabiodiversity.org/species/show/281378) | 2024 | accepted by WFO (placed as the accepted name of a taxon) |
| 381 | *Impatiens stolonifera* Robi & Manudev | YES | NO | YES | India | Manudev et al. | 2017 | accepted by WFO (placed as the accepted name of a taxon) |
| 382 | *Impatiens suksathanii* Ruchis. & Triboun | YES | NO | NO | Thailand | Ruchisansakun and Triboun | 2014 | accepted by WFO (placed as the accepted name of a taxon) |
| 383 | *Impatiens suichangensis* Y.L.Xu & Y.L.Chen | YES | NO | YES | China | Xu and Chen | 1999 | accepted by WFO (placed as the accepted name of a taxon) |
| 384 | *Impatiens suichangensis* Y.L.Xu & Y.L.Chen | YES | NO | YES | China | eFloras of China  (http://www.efloras.org/florataxon.aspx?flora_id=2&taxon_id=242326717) | 2008 | accepted by WFO (placed as the accepted name of a taxon) |
| 385 | *Impatiens sylvicola* Burtt Davy & Greenway | YES | NO | NO | Africa (country?) | Grey-Wilson | 1980 | accepted by WFO (placed as the accepted name of a taxon) |
| 386 | *Impatiens taihmushkulni* Chhabra & Ramneek | YES | YES | NO | India | Chhabra et al. | 2016 | accepted by WFO (placed as the accepted name of a taxon) |
| 387 | *Impatiens talbotii* Hook.f. | YES | NO | YES | India | Dessai and Janarthanam | 2008 | accepted by WFO (placed as the accepted name of a taxon) |
| 388 | *Impatiens talbotii* Hook.f. | YES | NO | YES | India | India Biodiversity Flora  (https://indiabiodiversity.org/species/show/264388?lang=en) | 2024 | accepted by WFO (placed as the accepted name of a taxon) |
| 389 | *Impatiens tangachee* Bedd. | YES | NO | NO | India | Beddome | 1859 | accepted by WFO (placed as the accepted name of a taxon) |
| 390 | *Impatiens tangachee* Bedd. | YES | NO | NO | India | Indian Biodiversity Portal  (https://indiabiodiversity.org/species/show/253692) | 2024 | accepted by WFO (placed as the accepted name of a taxon) |
| 391 | *Impatiens tayemonii* Hayata | YES | NO | YES | China | Huang | 1973 | accepted by WFO (placed as the accepted name of a taxon) |
| 392 | *Impatiens tayemonii* Hayata | YES | NO | NO | China | Chen et al.  eFloras of China (Flora of China)  (http://www.efloras.org/florataxon.aspx?flora_id=2&taxon_id=242326719) | 2008 | accepted by WFO (placed as the accepted name of a taxon) |
| 393 | *Impatiens teneriflora* Hook.f. | YES | NO | NO | India | ^ Hooker | 1910 | accepted by WFO (placed as the accepted name of a taxon) |
| 394 | *Impatiens teneriflora* Hook.f. | YES | NO | NO | India | India Biodiversity Portal  (https://indiabiodiversity.org/species/show/281382) | 2024 | accepted by WFO (placed as the accepted name of a taxon) |
| 395 | *Impatiens teysmanni* Miq. | YES | NO | NO | Indonesia | Miquel | 1860 | unplaced by WFO (a taxonomist hasn’t yet placed the name in the taxonomy), found on IPNI |
| 396 | *Impatiens theuerkaufiana* Ratheesh & Sivad. | YES | NO | YES | India | Narayanan et al. | 2013 | accepted by WFO (placed as the accepted name of a taxon) |
| 397 | *Impatiens theuerkaufiana* Ratheesh & Sivad. | YES | NO | NO | India | India Biodiversity Portal  <https://indiabiodiversity.org/species/show/281383> | 2024 | accepted by WFO (placed as the accepted name of a taxon) |
| 398 | *Impatiens thiochroa* Hand.-Mazz. | YES | NO | NO | China | Handel-Mazzetti | 1933 | accepted by WFO (placed as the accepted name of a taxon) |
| 399 | *Impatiens thiochroa* Hand.-Mazz. | YES | NO | NO | China | eFloras (Flora of China)  (http://www.efloras.org/florataxon.aspx?flora_id=2&taxon_id=242445589) | 2008 | accepted by WFO (placed as the accepted name of a taxon) |
| 400 | *Impatiens thoreli* Hook.f. | YES | NO | NO | Laos | #Hooker | 1909 | unplaced by WFO (a taxonomist hasn’t yet placed the name in the taxonomy) found on IPNI |
| 401 | *Impatiens thoreli* Hook.f. | YES | NO | YES | Indo-China | ****Hooker | 1910 | unplaced by WFO (a taxonomist hasn’t yet placed the name in the taxonomy) found on IPNI |
| 402 | *Impatiens thulunadensis* Sindhu Arya, Biju & V.S.A.Kumar | YES | YES | YES | India | Biju et al. | 2022 | accepted by WFO (placed as the accepted name of a taxon) |
| 403 | *Impatiens tianlinensis* S.X.Yu & L.J.Zhang | YES | NO | NO | China | Zeng et al. | 2015 | accepted by WFO (placed as the accepted name of a taxon) |
| 404 | *Impatiens tinctoria* A.Rich. | YES | NO | NO | Africa (country?) | Grey-Wilson | 1980 | accepted by WFO (placed as the accepted name of a taxon) |
| 405 | *Impatiens tigrina* Suksathan & Triboun | YES | NO | NO | Thailand | Suksathan and Triboun | 2009 | accepted by WFO (placed as the accepted name of a taxon) |
| 406 | *Impatiens tirunelvelliensis* L.Joseph & Bhaskar | YES | NO | NO | India | India Biodiversity Portal  (https://indiabiodiversity.org/species/show/281385) | 2024 | accepted by WFO (placed as the accepted name of a taxon) |
| 407 | *Impatiens tomentosa* B. Heyne | YES | NO | NO | India | Arigela et al. | 2022 | accepted by WFO (placed as the accepted name of a taxon) |
| 408 | *Impatiens tomentosa* B. Heyne | YES | NO | NO | India | India Biodiversity Portal  (https://indiabiodiversity.org/species/show/230023) | 2024 | accepted by WFO (placed as the accepted name of a taxon) |
| 409 | *Impatiens toppinii* Dunn | YES | NO | NO | Myanmar | Toppin | 1920 | accepted by WFO (placed as the accepted name of a taxon) |
| 410 | *Impatiens torulosa* Hook.f | YES | NO | NO | China | ***Hooker | 1908 | accepted by WFO (placed as the accepted name of a taxon) |
| 411 | *Impatiens tubifera* Hook.f. | YES | NO | NO | India | India Biodiversity Portal  (https://indiabiodiversity.org/species/show/281390) | 2024 | synonym of [***Impatiens scitula***](https://powo.science.kew.org/taxon/urn:lsid:ipni.org:names:374340-1) Hook.f.; accepted name in the genus *Impatiens* |
| 412 | *Impatiens turrialbana* Donn.Sm. | YES | NO | NO | Panamá | Elias | 1967 | accepted by WFO (placed as the accepted name of a taxon) |
| 413 | *Impatiens tribounni* T.Shimizu & Suksathan | YES | NO | NO | Thailand | Shimizu and Suksathan | 2004 | not found on WFO, found on IPNI |
| 414 | *Impatiens trichopoda* Hook.f. | YES* | NO | NO | China | Hooker | 1908 | accepted by WFO (placed as the accepted name of a taxon) |
| 415 | *Impatiens trichopoda* Hook.f. | YES* | NO | NO | China | Chen et al.  eFloras (Flora of China)  (http://www.efloras.org/florataxon.aspx?flora_id=2&taxon_id=242445596) | 2008 | accepted by WFO (placed as the accepted name of a taxon) |
| 416 | *Impatiens trichocarpa* Hook.f. | YES | NO | YES | India | ****Hooker | 1910 | accepted by WFO (placed as the accepted name of a taxon) |
| 417 | *Impatiens tweedieae* E.A.Bruce | YES | NO | NO | Africa (country?) | Grey-Wilson | 1980 | accepted by WFO (placed as the accepted name of a taxon) |
| 418 | *Impatiens umbellata* B.Heyne | YES | NO | NO | India | Wight | 1837 | accepted by WFO (placed as the accepted name of a taxon) |
| 419 | *Impatiens umbellata* B.Heyne | YES | NO | NO | India | India Biodiversity Portal  (https://indiabiodiversity.org/species/show/253695) | 2024 | accepted by WFO (placed as the accepted name of a taxon) |
| 420 | *Impatiens uncinata* Wight | YES | NO | YES | India | Wight | 1837 | accepted by WFO (placed as the accepted name of a taxon) |
| 421 | *Impatiens uncinata* Wight | YES | NO | NO | India | Indian Biodiversity Portal  (https://indiabiodiversity.org/species/show/253693) | 2024 | accepted by WFO (placed as the accepted name of a taxon) |
| 422 | *Impatiens uniflora* Hayata | YES | NO | NO | Taiwan | Hayata | 1908 | accepted by WFO (placed as the accepted name of a taxon) |
| 423 | *Impatiens uniflora* Hayata | YES | NO | YES | Taiwan | Huang | 1973 | accepted by WFO (placed as the accepted name of a taxon) |
| 424 | *Impatiens uniflora* Hayata | YES | NO | NO | Taiwan | Chen et al.  eFloras of China (Flora of China)  (http://www.efloras.org/florataxon.aspx?flora_id=2&taxon_id=200013309) | 2008 | accepted by WFO (placed as the accepted name of a taxon) |
| 425 | *Impatiens usambarensis* Grey-Wilson | YES | NO | NO | Tanzania | Grey-Wilson | 1980 | accepted by WFO (placed as the accepted name of a taxon) |
| 426 | *Impatiens vagans* Hook.f. | YES | NO | NO | Cambodia | #Hooker | 1909 | accepted by WFO (placed as the accepted name of a taxon) |
| 427 | *Impatiens vagans* Hook.f. | YES | NO | YES | Cambodia | ****Hooker | 1910 | accepted by WFO (placed as the accepted name of a taxon) |
| 428 | *Impatiens velaxata* Hook.f. | YES | NO | NO | Cambodia | #Hooker | 1909 | accepted by WFO (placed as the accepted name of a taxon) |
| 429 | *Impatiens veerapazhasii* Ratheesh, Sujanapal & Meera | YES | NO | YES | India | Kumar et al. | 2011 | accepted by WFO (placed as the accepted name of a taxon) |
| 430 | *Impatiens veerapazhasii* Ratheesh, Sujanapal & Meera | YES | NO | YES | India | India Biodiversity Portal  (https://indiabiodiversity.org/species/show/263016) | 2024 | accepted by WFO (placed as the accepted name of a taxon) |
| 431 | *Impatiens verecunda* Hook.f. | YES | NO | NO | India | ********* Hooker | 1911 | accepted by WFO (placed as the accepted name of a taxon) |
| 432 | *Impatiens verecunda* Hook.f. | YES | NO | NO | India | India Biodiversity Portal  (https://indiabiodiversity.org/species/show/263017) | 2024 | accepted by WFO (placed as the accepted name of a taxon) |
| 433 | *Impatiens verrucifer* Hook.f. | YES | NO | YES | Vietnam | *Hooker | 1908 | accepted by WFO (placed as the accepted name of a taxon) |
| 434 | *Impatiens vexillaria* Hook.f. | YES | NO | NO | India | Indian Biodiversity Portal  (<https://indiabiodiversity.org/species/show/281392)> | 2024 | accepted by WFO (placed as the accepted name of a taxon) |
| 435 | *Impatiens viscida* Wight | YES | NO | NO | India | Wight | 1837 | accepted by WFO (placed as the accepted name of a taxon) |
| 436 | *Impatiens viscida* Wight | YES | NO | NO | India | India Biodiversity Portal  (https://indiabiodiversity.org/species/show/253746) | 2024 | accepted by WFO (placed as the accepted name of a taxon) |
| 437 | *Impatiens viscosa* Bedd. | YES | NO | NO | India | Beddome | 1859 | accepted by WFO (placed as the accepted name of a taxon) |
| 438 | *Impatiens viscosa* Bedd. | YES | NO | NO | India | Utami and Shimizu | 2005 | accepted by WFO (placed as the accepted name of a taxon) |
| 439 | *Impatiens viscosa* Bedd. | YES | NO | NO | India | India Biodiversity  (https://indiabiodiversity.org/species/show/253744) | 2024 | accepted by WFO (placed as the accepted name of a taxon) |
| 440 | *Impatiens violacea* M.Kumar & Sequiera | YES | NO | YES | India | Kumar and Sequiera | 2001 | accepted by WFO (placed as the accepted name of a taxon) |
| 441 | *Impatiens violacea* M.Kumar & Sequiera | YES | NO | NO | India | India Biodiversity Flora  (https://indiabiodiversity.org/species/show/263018) | 2024 | accepted by WFO (placed as the accepted name of a taxon) |
| 442 | *Impatiens volkensii* Warb. | YES | NO | NO | Tanzania | Grey-Wilson | 1980 | accepted by WFO (placed as the accepted name of a taxon) |
| 443 | *Impatiens violascens* B.U.Oh & Y.Y.Kim | YES | YES | YES | Korea | Kim | 2010 | accepted by WFO (placed as the accepted name of a taxon) |
| 444 | *Impatiens wallichii* Hook.f. | YES | NO | NO | India | Indian Biodiversity Portal  (https://indiabiodiversity.org/species/show/281395) | 2024 | accepted by WFO (placed as the accepted name of a taxon) |
| 445 | *Impatiens wattii* Hook.f. | YES | NO | NO | India | Indian Biodiversity Portal  (https://indiabiodiversity.org/species/show/281396) | 2024 | accepted by WFO (placed as the accepted name of a taxon) |
| 446 | *Impatiens winkleri* Hook.f. | YES | NO | NO | Borneo | %Hooker | 1910 | accepted by WFO (placed as the accepted name of a taxon) |
| 447 | *Impatiens williamsii* H.Hara | YES | NO | NO | Nepal | Sherpa et al. | 2021 | accepted by WFO (placed as the accepted name of a taxon) |
| 448 | *Impatiens wolfgangii* J.Lali & Bhaskar | YES | YES | NO | India | Lali and Bhaskar | 2017 | accepted by WFO (placed as the accepted name of a taxon) |
| 449 | *Impatiens x pacifica* Zika | YES | NO | YES | USA | Zika | 2006 | accepted by WFO (placed as the accepted name of a taxon) |
| 450 | *Impatiens wightiana* Bedd. | YES | NO | NO | India | Beddome | 1859 | not found on WFO, found on IPNI |
| 451 | *Impatiens wightiana* Bedd. | YES | NO | NO | India | India Biodiversity Portal  (https://indiabiodiversity.org/species/show/255668) | 1859 | not found on WFO, found on IPNI |
| 452 | *Impatiens wrayi* Hook | YES | NO | NO | Malaysia | ***Hooker | 1909 | accepted by WFO (placed as the accepted name of a taxon) |
| 453 | *Impatiens yaoshanensis* K.M.Liu & Y.Y.Cong | YES | NO | YES | China | Cong et al. | 2008 | accepted by WFO (placed as the accepted name of a taxon) |
| 454 | *Impatiens yercaudensis* Bhaskar | YES | NO | NO | India | (https://indiabiodiversity.org/species/show/281398) | 2024 | accepted by WFO (placed as the accepted name of a taxon) |
| 455 | *Impatiens yercaudensis* Bhaskar | YES | NO | NO | India | India Biodiversity Portal  (https://indiabiodiversity.org/species/show/281398) | 2024 | accepted by WFO (placed as the accepted name of a taxon) |
| 456 | *Impatiens veerapazhasii* Ratheesh, Sujanapal & Meera | YES | NO | YES | India | Kumar et al. | 2011 | accepted by WFO (placed as the accepted name of a taxon) |
| 457 | *Impatiens veerapazhasii* Ratheesh, Sujanapal & Meera | YES | NO | YES | India | India Biodiversity Portal  (https://indiabiodiversity.org/species/show/263016) | 2024 | accepted by WFO (placed as the accepted name of a taxon) |
| 458 | *Impatiens violaeflora* Hooker Burma | YES | NO | NO | Burma | Toppin | 1920 | not found on WFO or IPNI |
| 459 | *Impatiens yingjingensis* [Xin Q.Song](https://www.ipni.org/a/20074379-1), [B.N.Song](https://www.ipni.org/a/20074380-1) & [Biao Yang](https://www.ipni.org/a/20074381-1), | YES | YES | YES | China | Song et al. | 2024 | unplaced by WFO (a taxonomist hasn’t yet placed the name in the taxonomy), found on IPNI |
| 460 | *Impatiens yinyinkyii* Latt, B.B.Park & Nob.Tanaka | YES | YES | NO | Myanmar | Latt et al. | 2023 | unplaced by WFO (a taxonomist hasn’t yet placed the name in the taxonomy), found on IPNI |
| 461 | *Impatiens zhuxiensis* Q.L.Gan & X.W.Li | YES | YES | NO | China | Gan and Li | 2020 | accepted by WFO (placed as the accepted name of a taxon) |
| 462 | *Impatiens zombensis* Baker | YES | NO | NO | Africa (country) | Grey-Wilson | 1980 | accepted by WFO (placed as the accepted name of a taxon) |

**References**

Andrews FW (1950) The flowering plants of the Anglo-Egyptian Sudan. Vol. 1. (Cycadaceae-Tiliaceae). Published for the Sudan Government by T. Buncle & Co., Ltd., Arbroath, Scotland, 237 pp.

Arigela RK, Kabeer KAA (2022). New synonyms in three southern Indian endemic species of *Impatiens* (Balsaminaceae). Annales Botanici Fennici 59(1): 261–271. <https://doi.org/10.5735/085.059.0138>

Baillon HE (1891) Volume XXIX. Histoire naturelle des plantes. In: Grandidier A (Eds) Histoire physique, naturelle et politique de Madagascar, tome III – Atlas – II, 2e partie, fasc. 27. Imprimerie Nationale, Paris.

Beddome RH (1859) The genus *Impatiens*. Description of new species from the Anamalai hills. Madras Journal of Literature and Science 2 4(7): 66–74.

Beddome RH (1864) Contributions to the botany of Southern India by Captain R. H. Bedomee officiating Conservator of Forests, with Plates. Madras Journal of Literature and Science 3(1): 37–59

Bhaskar V (2006) *Impatiens clavata* Bhaskar, a new scapigerous balsam (Balsaminaceae) from Bisle Ghat, Western Ghats, South India. Current Science 91(9): 1138–1140.

Bhaskar V, Razi BA (1982) Two more new species of *Impatiens* L. from South India. Journal of Bombay National History Society 79: 382–384.

Bhaskar V. (2012) Taxonomic Monograph on Impatiens L. (Balsaminaceae) of Western Ghats, South India: The Key Genus for Endemism. Centre for plant taxonomic studies, Bangalore, 283 pp.

Biju P, Arya S, Augustine J, Josekutty EJ, Kumar VNSA (2022) *Impatiens thulunadensis* (Balsaminaceae), a new species from northern Kerala, India. Annales Botanici Fennici 59(1): 207–212. [https://](https://euc-word-edit.officeapps.live.com/we/wordeditorframe.aspx?ui=pl-PL&rs=pl-PL&wopisrc=https%3A%2F%2Funiwersytetlodzki-my.sharepoint.com%2Fpersonal%2Fagnieszka_stefaniak_biol_uni_lodz_pl%2F_vti_bin%2Fwopi.ashx%2Ffiles%2F6ee0c659bbc94839aac626d651c93e9a&wdenableroaming=1&wdfr=1&mscc=1&wdodb=1&hid=67962BA1-B034-8000-F44B-4707B2AE9E12.0&uih=sharepointcom&wdlcid=pl-PL&jsapi=1&jsapiver=v2&corrid=d66c44e1-6471-d787-92c6-0c306a1d65e7&usid=d66c44e1-6471-d787-92c6-0c306a1d65e7&newsession=1&sftc=1&uihit=docaspx&muv=1&cac=1&sams=1&mtf=1&sfp=1&sdp=1&hch=1&hwfh=1&dchat=1&sc=%7B%22pmo%22%3A%22https%3A%2F%2Funiwersytetlodzki-my.sharepoint.com%22%2C%22pmshare%22%3Atrue%7D&ctp=LeastProtected&rct=Normal&wdorigin=Other&instantedit=1&wopicomplete=1&wdredirectionreason=Unified_SingleFlush)doi: [10.5735/085.059.0132](http://dx.doi.org/10.5735/085.059.0132)

Biju SD (2001) Relocation of *Impatients anaimudica* CEC Fisch. (Balsaminaceae) and the taxonomic status of *I. konalarensis* Chandrab. et al. Rheedea-kerala 11(2): 109–113.

Bojňanský V, Fargašová A (2007) Atlas of seeds and fruits of Central and East-European flora. The Carpathian Mountains region. Springer, Dordrecht, 1046 pp.

Cheek M, Csiba L (2002) A new epiphytic species of *Impatiens* (Balsaminaceae) from western Cameroon. Kew Bulletin 57(3): 669–674. https://doi.org/10.2307/4110997

Cheek M, Osborne J, van der Burgt X, Darbyshire I, Onana JM (2023) *Impatiens banen* and *Impatiens etugei* (Balsaminaceae), new threatened species from lowland of the Cross-Sanaga Interval, Cameroon. Kew Bulletin 78: 67–82. https://doi.org/10.1007/s12225-022-10073-w

Chen Y, He BQ, Li MJ, Huang R, Bai XX (2022) *Impatiens nayongensis* (Balsaminaceae), a new species from Guizhou, China. ARPHA Preprints 3: e95944. https:// doi: 10.3897/arphapreprints.e95944

Chen YL (1980) Taxa nova Balsaminacearum e flora Xizangensi (Tibetica). Acta Phytotaxonomica Sinica 18(3): 380–382

Chen Y-L (1999) Six new species of *Impatiens* L. from China. Journal of Systematics and Evolution 37(1): 88–99.

Chen Y-L (2000) Three new species of *Impatiens* L. from China. Journal of Systematics and Evolution 38(6): 557–562.

ChenYL, Akiyama S, Ohba H (2008). Balsaminaceae. In: Wu Z, Raven PH, Hong DY (Eds) Flora of China, vol. 12. Science Press, Beijing & Missouri Botanical Garden Press, St. Louis, USA, 43–144.

Chhabra T, Singh R, Prabhukumar KM, Hareesh VS (2016) Three new taxa of *Impatiens* (Balsaminaceae) from southern Western Ghats, India. Nordic Journal of Botany 34(6): 708–717. https://doi.org/10.1111/njb.01139

Cong YY, Liu KM, Tian SZ (2008) *Impatiens yaoshanensis* (Balsaminaceae), a new species from Yunnan, China. Annales Botanici Fennici 45(2): 148–150. <https://doi.org/10.5735/085.045.0210>

Dalzell NA (1851) Contributions to the botany of Western India. Hooker’s Journal of Botany and Kew Garden Miscellany 3: 206–212.

Dessai JR, Janarthanam MK (2008) Taxonomy and distribution of *Impatiens talbotii*: A rare endemic balsam from Western Ghats. Rheedea 21(2011): 23–80.

Dessai JRN, Joseph L, Janarthanam MK (2009) A new species of epiphytic *Impatiens* (Balsaminaceae) from the Western Ghats, India. Taiwania 54(2): 149–151.

Douglas GW, Straley GB, Meidinger D, Pojar J (1998) Illustrated Flora of British Columbia, Volume 2, Dicotyledons (Balsaminaceae through Cuscutaceae). Vol 2. British Columbia Ministry of Environment, Lands and Parks, Ministry of Forests: Victoria, BC, Canada, 1–401.

Edgeworth MP (1846) III. Descriptions of some unpublished Species of Plants from North‐Western India. Transactions of the Linnean Society of London 20(1): 23–91.

eFloras (Flora of China) (2008) *Balsaminaceae* <http://www.efloras.org/florataxon.aspx?flora_id=2&taxon_id=116392> [accessed 20.10.2024]

Elias TS (1967) Part VI. Family 110: Balsaminaceae. In Woodson RE, Jr Schery RW (Eds) Flora of Panama Volume 54. Annals of the Missouri Botanical Garden; Missouri Botanical Garden Press: St. Louis, MO, USA, 21–24.

Fischer CEC (1931) Plants New to Assam: III. Bulletin of Miscellaneous Information (Royal Botanic Gardens, Kew) 1931(5): 281–285. <https://doi.org/10.2307/4102490>

Fischer CEC (1934) New or Little-Known Plants from South India: IV. Bulletin of Miscellaneous Information (Royal Botanic Gardens, Kew) 1934(9): 389–394. https://doi.org/10.2307/4115431

Fischer E, Rahelivololona ME (2007) New taxa of *Impatiens* from Madagascar IV. Adansonia 29(2): 269–315

Franchet A (1886) Bulletin de la Société botanique de France. La Société, Paris. 33: 447.

Fujimoto Y, Tagane S, Rabarison H, Andriamahasetra RN, Komada N, Kitamura K (2024) *Impatiens alboarenicola*, a new species of Balsaminaceae from north-western Madagascar. Phytotaxa 650(3): 206–212. <https://doi.org/10.11646/phytotaxa.650.3.2>

Gan Q, Li X (2020) *Impatiens zhuxiensis* (Balsaminaceae), a new species of Hubei, China. Nordic Journal of Botany 38(4): e02686. https://doi.org/10.1111/njb.02686

Gan QL, Li XW (2016) *Impatiens baokangensis* (Balsaminaceae), a new species from Hubei, China. Annales Botanici Fennici 53(3/4): 145–148. <https://doi.org/10.5735/085.053.0401>

=Gogoi R, Borah S (2015) *Impatiens ashihoi*, a new species of Balsaminaceae from Dibang Valley of Arunachal Pradesh, India. Phytotaxa 238(3): 278–282. <https://doi.org/10.11646/phytotaxa.238.3.7>

Gogoi R, Borah S (2015) *Impatiens adamowskiana* sp. nov. (Balsaminaceae) from Arunachal Pradesh, northeast India. Nordic Journal of Botany 33(5): 586–590. <https://doi.org/10.1111/njb.00798>

Gogoi R, Borah S (2015) *Impatiens dalaiensis* (Balsaminaceae) a new species from Arunachal Pradesh, India. Phytotaxa 207(3): 286–290. <https://doi.org/10.11646/phytotaxa.207.3.7>

%%Gogoi R, Borah S (2015) *Impatiens pathakiana* (Balsaminaceae), a new species from Arunachal Pradesh, north-eastern India. Telopea 18: 121–125. https://doi.org/10.7751/telopea8484

Gogoi R, Kumar A, Adamowski W (2018) Notes on *Impatiens sikkimensis* (Balsaminaceae) and its recollection after a century. Nelumbo 60(1): 23–25. <https://doi.org/10.20324/nelumbo/v60/2018/130179>

Gogoi R, Sherpa N, Borah S (2020) Taxonomic note on *Impatiens longipes* (Balsaminaceae) and description of a new species, *I. pseudolongipes*. Phytotaxa 429(3): 217–224. <https://doi.org/10.11646/phytotaxa.429.3.3>

Gogoi, R, Borah S (2013) *Impatiens lohitensis,* a New Species of *Impatiens* (Balsaminaceae) from Arunachal Pradesh, India. Taiwania 58(1): 15–19. https://doi.org/10.6165/tai.2013.58.15

Grey-Wilson C (1980) *Impatiens of Africa*. CRC Press, Rotterdam, The Netherlands, 235 pp.

Hallé N (1962) Mélianthacées, balsaminacées, rhamnacées. Flore du Gabon. National Museum of Natural History Paris 4: 1–74.

Handel-Mazzetti (1933) Balsaminaceae V.F. Brotherus, et al. (Eds.), Symbolae sinicae: botanische Ergebnisse der Expedition der Akademie der Wissenschaften in Wein nach Südwest-China, 1914–1918, Julius Springer, Vienna (1933), pp. 538-539

Hareesh VS, Sreekumar VB, Dantas, KJ, Sujanapal P (2015). *Impatiens sahyadrica* sp. nov. (Balsaminaceae)—a new species from southern Western Ghats, India. Phytotaxa 207(3): 291–296. https://doi.org/10.11646/phytotaxa.207.3.8

Hasskarl JK (1858) Hortus Bogoriensis descriptus: sive retziae editio nova valda aucta et emendata. Pars prima. F. Günst, Amsterdam.

Hayata B (1908) Flora montana formosae; an enumeration of plants found on Mt. Morrison, the central chain and other mountainous regions of Formosa at altitudes of 3,000-13,000 ft. Journal of the College of Science, Imperial University of Tokyo, Japan 25(19): 1–260. <https://doi.org/10.5962/bhl.title.10880>

Hooker (1910) Icones plantarum or figures, with brief descriptive characters and remarks, of new or rare plants, selected from the Kew herbarium Fourth series Vol X. London, Longman, Rees. https://doi.org/10.5962/bhl.title.100719

Hooker (1911) Icones plantarum or figures, with brief descriptive characters and remarks, of new or rare plants, selected from the Kew herbarium Fourth series Vol X. London, Longman, Rees. https://doi.org/10.5962/bhl.title.100719

***Hooker J (1908) Les especes du genre ‘*Impatiens*’ dans l'herbier du Museum de Paris. Nouvelles Archives du Muséum d'Histoire Naturelle Paris 10: 233–272

********Hooker JD (1863) On the Plants of the Temperate Regions of the Cameroons Mountains and Islands in the Bight of Benin; collected by Mr. Gustav Mann, Government Botanist. Journal of the Proceedings of the Linnean Society of London. Botany 7(28): 171–240. <https://doi.org/10.1111/j.1095-8312.1864.tb01067c.x>

?Hooker JD (1875–1897) The flora of British India, vol 1–7. L. Reeve and Co., London, 1–740.

******Hooker JD (1909) A review of the known Philippine Islands species of *Impatiens*. Bulletin of Miscellaneous Information (Royal Botanic Gardens, Kew) 1909(7): 281–289.

#Hooker JD (1909) *Impatiens* d’Indo-Chine. In Notulae Systematicae, Laboratoire de Phanérogamie, Vol. 1. Lecomte, H. (ed.), Muséum National d’Histoire Naturelle, Paris, 10–14.

}Hooker JD (1909) On some species of Impatiens from Indo-China and the Malayan peninsula. Bulletin of Miscellaneous Information, Royal Gardens, Kew, 1: 1–12

^Hooker JD (1910) Indian species of *Impatiens*: generis *Impatiens* species Indicae novae et minus rite cognitae a cl. A. Meebold. Bulletin of Miscellaneous Information, Kew 1910: 291–300

%Hooker JD (1910) XIII. DECADES KEWENSES Plantarum Novarum in Herbario Horti Regii Conservatarum. DECAS LVI. Bulletin of Miscellaneous Information, Kew 3: 73–79

**Hooker JD (1910) XXXVIII, New *Impatiens* from China. Bulletin of Miscellaneous Information (Royal Botanic Gardens, Kew) 1910: 269–274.

*******Hooker JD (1911) Indian species of *Impatiens*. On some Western Peninsular Indian Balsamineae collected by Mr. A. Meebold. Bulletin of Miscellaneous Information Royal Botanic Gardens, Kew 1911(9): 353–356.

*Hooker WJ (1908) Icones plantarum: or figures, with brief descriptive characters and remarks, of new or rare plants, selected from the author's herbarium Fourth series (Vol. 29) Part III. Dulau & Co 37 Soho Square, London, 1–245.

Hooker JD, Thompson T (1859) Praecursores a Indicam. Balsaminaceae. [Journal of the Proceedings of the Linnean Society of London. Botany](https://onlinelibrary.wiley.com/journal/19459424) 4(15): 106–157. <https://doi.org/10.1111/j.1095-8339.1859.tb01160.x>

Huang RX, He BQ, Chen Y, Li MJ, Bai XX (2023) *Impatiens cavaleriei* (Balsaminaceae), a new species from the Miaoling Mountains in Guizhou. Taiwania 68(1): 85–89. https://doi.org/10.6165/tai.2023.68.85

Huang TC (1973) A revision of Formosan *Impatiens* (Balsaminaceae). Taiwania 18(1) 49–54.

Humbert H (1956) Contributions à l'étude de la flore de Madagascar et des Comores (fascicule 5). Notulae Systematicae (Paris) 15: 113–134.

India Biodiversity Portal (2024). Genus *Impatiens*. [https://indiabiodiversity.org/species/list?max=16&offset=0&sort=species.lastUpdated&taxon=113641&userGroupList&view=grid]

Joe A, Sabu, M (2017) *Impatiens agastyamalayensis* stat. Nov .(Balsaminaceae). A reassessment of *Impatiens* *rufescens* var. *agastyamalayensis* and rediscovery of the plant from the Western Ghats. *Phytotaxa* 326:144–150. https://doi.org/10.11646/phytotaxa.326.2.6

Kale PV, Shinde RD (2023) Lectotypification of *Impatiens kleiniformis* Sedgw. (Balsaminaceae) and Habenaria multicaudata Sedgw. (Orchidaceae)- The two endemic plant species described by LJ Sedgwick from Southern-Western Indian Peninsula. Journal of Global Biosciences 12(3): 9735–9746

Kim YY, Ji SJ, Hong WP, Oh BU (2010) *Impatiens violascens* (Balsaminaceae): A new species from Korea. Korean Journal of Plant Taxonomy 40(1): 59–64. <https://doi.org/10.11110/kjpt.2010.40.1.059>

Kumar M, Sequiera S (2001) Two new species of *Impatiens* (Balsaminaceae) from India. SIDA, Contributions to Botany 19(1): 795–801.

Kumar M, Sequiera S (2001) Two new species of *Impatiens* (Balsaminaceae) from India. SIDA*,* Contributions to Botany 19(4): 795–801.

Kumar NA, Narayanan, MR, Sujanapal P, Raj RM, Sujana KA, Mithunlal KA (2011) *Impatiens veerapazhasii* (Balsaminaceae), a new scapigerous balsam from Wayanad, Western Ghats, India. Journal of the Botanical Research Institute of Texas 5(1): 153–158.

Lali J, Bhaskar V (2017) *Impatiens wolfgangii* – a new scapigerous balsam (Balsaminaceae) from Bababudangiri, Western Ghats, India. Phytotaxa 313(3): 281–284. <https://doi.org/10.11646/phytotaxa.313.3.6>

Latt MM, Tanaka N, Park BB (2023) Two New species of *Impatiens* (Balsaminaceae) from Myanmar. Phytotaxa 583(2):141–152. <https://doi.org/10.11646/phytotaxa.583.2.2>

Launert E (1962) New and little-known species from the Flora Zambesiaca area. Boletim da Sociedade Broteriana 36: 47–65.

Lu ZC, Pan B, Huang FZ, Liu Y (2020) *Impatiens gongchengensis* (Balsaminaceae), a new species from Guangxi, Southern China. Taiwania 65(1): 1–4. <https://doi.org/10.6165/tai.2020.65.1>

Luo Q (2011) *Impatiens liangshanensis* Q. Luo, a new species of *Impatiens* (Balsaminaceae) from Sichuan, China. Guihaia 31: 433-435.

Makino T (1911) Observations on the Flora of Japan (Continued from p. 18.). Shokubutsugaku Zasshi 25(293): en153-en158.

Mani B, Thomas S (2017) *Impatiens brittoi* (Balsaminaceae) sp. nov. from the southern Western Ghats, India. Nordic Journal of Botany 35(4): 440–444. <https://doi.org/10.1111/njb.01485>

Mani B, Thomas S, Britto SJ (2018) Two new species of *Impatiens* (Balsaminaceae) from the Western Ghats, India. Phytotaxa 334(3): 233–240. https://doi.org/10.11646/phytotaxa.334.3.4.

Mani B, Thomas S, Britto SJ (2020) A new species of *Impatiens* (Balsaminaceae) and rediscovery of *Impatiens aliciae* from the Western Ghats of India. Taiwania 65(4): 451–455.

Manudev KM, Robi AJ, Nampy S (2017) *Impatiens stolonifera* (Balsaminaceae): A new scapigerous species from the southern Western Ghats, Kerala, India. Phytotaxa 295(1): 71–76. <https://doi.org/10.11646/phytotaxa.295.1.6>

Miquel FAW 1860 Diagnoses specierum et generum novorum ordinumque, in Florae In: van der Post CG, van der Post C, (Eds) [Flora van Nederlandsch Indië, Eerste Bijvoegsel](https://scholar.google.com/scholar_lookup?title=Flora%20van%20Nederlandsch%20Indie,%20Eerste%20Bijvoegsel%20Supplement&author=van%20der%20Post&publication_year=1860) Sumatra, Zijne Plantenwfreld En Hare Voortbrengselen. Met Platen. Leipzig: bij Fried Fleischer, 280–617.

Monzalvo R, Escorcia-Guerrero DL, García-Montes MA, Rewicz A, Rewicz T, Manríquez-Morán NL (2024) The Mexican Balsam, *Impatiens mexicana* Rydb: A Redescription Based on Morphological and Phylogenetic Studies, with an Update of the Current Geographical Range of the Species. Diversity 16(2): 87. <https://doi.org/10.3390/d16020087>

°Narayanan MR, Kumar NA, Joseph JP, Sunil C.N, Shaju T (2012) *Impatiens johnsiana* (Balsaminaceae), A new scapigerous balsam from Western Ghats, India. Journal of the Botanical Research institute of Texas 6(1): 113–118.

Narayanan MKR, Jayesh PJ, Kumar A, Sivadasan M, Alfarhan A (2013) *Impatiens theuerkaufiana* (Balsaminaceae), a new scapigerous species from the Western Ghats, India. Phytotaxa 83(1): 54–60. https://doi.org/10.11646/phytotaxa.83.1.3

Narayanan MR, Sujana KA, Balakrishnan V, Raj RM, Kumar NA (2012) *Impatiens mohana* (Balsaminaceae), a new scapigerous balsam from Wayanad, Western Ghats, India. Edinburgh Journal of Botany 69(2): 281–285. <https://doi.org/10.1017/S096042861200008X>

Oh A, Jang HD, Lee JS, Oh BU (2022) *Impatiens hambaeksanensis* (Balsaminaceae), a new species from South Korea. PhytoKeys 211: 139–150. https://doi.org/10.3897/phytokeys.211.90236

Oh A, Oh BU, Oh HK (2024) *Impatiens jangjeonense* (Balsaminaceae), a new species from South Korea. Phytotaxa 663(5): 283–293.https://doi.org/10.11646/phytotaxa.663.5.3.

Pandurangan AG, Nair VJ (1995) *Impatiens kulamavuensis*, a new species of Balsaminaceae from India. Novon 5(1): 57–58. https://doi.org/10.2307/3391835

Prabhukumar KM, Hareesh VS, Bhaskar V, Sreekumar VB, Nirmesh TK, Balachandran I (2016) *Impatiens glabrata* (Balsaminaceae)—A new species from southern Western Ghats, India. Phytotaxa 266(1): 33–39. <https://doi.org/10.11646/phytotaxa.266.1.5>

Prabhukumar KM, Hareesh VS, Sreekumar VB, Nirmesh TK, Balachandran I (2015) *Impatiens neo-modesta* (Balsaminaceae) – a new species from Western Ghats, India. Webbia 70(2): 231–235. https://doi.org/10.1080/00837792.2015.1074792

Prabhukumar KM, Jagadeesan R, Kumar VN, Prasad G, Balachandran I (2018) Rediscovery of *Impatiens brevicornis* L. (Balsaminaceae), a less known species of Western Ghats, India. Nelumbo 60(1): 13–17. <https://doi.org/10.20324/nelumbo/v60/2018/118720>

¨Prabhukumar, KM, Jagadeesan, R, Prasad, G, Nagaraj, BANA, Kumar, VVN, Veeralinga B, Balachandran I (2015). Two new taxa of *Impatiens* (Balsaminaceae) from Western Ghats, India. Phytotaxa 238(3): 255–264. <https://doi.org/10.11646/phytotaxa.238.3.4>

Prabhukumar KM, Jagadeesan R, Prasad G, Nagaraj BANA, Kumar VVN, Veeralinga B, Balachandran I (2017) Two new taxa of *Impatiens* (Balsaminaceae) from southern parts of Western Ghats, India. Phytotaxa 296(3): 281–286. <https://doi.org/10.11646/phytotaxa.437.5.3>

Raju R, Dhanraj FI, Arumugam M, Pandurangan AG (2015) *Impatiens matthewiana*, a new scapigerous balsam from Western Ghats, India. Phytotaxa 227(3): 268–274. <https://doi.org/10.11646/phytotaxa.227.3.6>

Ramasubbu R, Anjana S, Prabha AC (2020) A new species *Impatiens* L. (Balsaminaceae) from Kodaikkanal Wildlife Sanctuary, India. Taiwania 65(4): 426–430.

Ramasubbu R, Divya C, Sasi Kala N, Anjana S, Sreekala AK (2017) *Impatiens megamalayana*, a new species of *Impatiens* from the Western Ghats, Tamil Nadu, India. Phytotaxa 302(2): 193–197. <https://doi.org/10.11646/phytotaxa.302.2.10>

Raskoti BB, Ale R (2022) A new species of *Impatiens* and updated checklist of Balsaminaceae in Nepal. Plos one 17(10): e0274699. https://doi.org/10.1371/journal.pone.0274699

Ren LY, Chen Y, Yuan TH, Huang RX, Li MJ, Bai XX (2022) *Impatiens bijieensis* (Balsaminaceae), a new species from karst plateau in Guizhou, China. PhytoKeys 192: 1–10. https://doi.org/10.3897/phytokeys.192.77517

Rewicz A, Adamowski W, Borah B, Gogoi R (2020) New Data on Seed Coat Micromorphology of Several *Impatiens* spp. from Northeast India. Acta Societatis Botanicorum Poloniae 89(3): 89312. https://doi: 10.5586/asbp.89312.

Richard PSS, Karuppusamy S, Ravichandran V (2022) *Impatiens godfreyi* (Balsaminaceae), a new species of Balsam from the southern Western Ghats, India. Journal of Asia-Pacific Biodiversity 15(1): 138–144. https://doi.org/10.1016/j.japb.2021.12.001

Richard PSS, Ravichandran V (2023) *Impatiens karuppusamyi* (Balsaminaceae), a New Species from the Southern Western Ghats, India. Annales Botanici Fennici 60(1): 131–135. https://doi.org/10.5735/085.060.0121

Richard PSS, Ravichandran V (2023) *Impatiens karuppusamyi* (Balsaminaceae), a new species from the southern Western Ghats, India. Annales Botanici Fennici 60(1): 131–135. <https://doi.org/10.5735/085.060.0121>

Roxburgh W (1824) Flora indica, or descriptions of Indian plants. Vol. 2 [Carey W, Wallich N, eds]. Mission Press, Serampore, 583 pp.

Ruchisansakun S, Triboun P, Jenjittikul T (2014) A new species of *Impatiens* (Balsaminaceae) from Southwestern Thailand. Phytotaxa 174(4): 237–241. https://doi.org/10.11646/phytotaxa.174.4.5

Saravanan TS, Kaliamoorthy S (2024) *Impatiens brahmagiriana* (Balsaminaceae): A new species from the southern Western Ghats of India. Taiwania 69: 1–64: [https://doi.org/10.6165/tai.2024.69.99](https://www.airitilibrary.com/Common/Click_DOI?DOI=10.6165%2ftai.2024.69.99)

Sherpa N, Kasaju SK, Subedi S, Gogoi R (2021) Notes on The Recollection and Typification of *Impatiens williamsii* H. Hara (Balsaminaceae) Little Known Endemic Species of Nepal. Nelumbo 63(2): 23–27. <https://doi.org/10.20324/nelumbo/v63/2021/167712>

Shimizu T (1969) Some new species from Thailand. Acta Phytotaxonomica et Geobotanica, 24(1-2): 35–42. <https://doi.org/10.18942/bunruichiri.KJ00001078054>

Shimizu T (1991) New species of the Thai *Impatiens* (1). Shokubutsu Kenkyu Zasshi 66(3): 166–171.

Shimizu T (2000) New species of the Thai *Impatiens* (Balsaminaceae): 2. Bulletin of the National Science Museum, Series B (Botany) 26(2): 35–42.

Shimizu T, Suksathan P (2004) Three new species of the Thai *Impatiens* (Balsaminaceae). Part 3. Bulletin of the National Science Museum, Series B, Botany 30(4): 165–171.

Smith WW (1914) Diagnoses Specierum Novarum in Herbario Horti Regii Botanici Edinburgensis Cognitarum LI-CII. Notes from the Royal Botanic Garden Edinburgh 8(38): 173–212

Smith WW (1915) Diagnoses Specierum Novarum in Herbario Horti Regii Botanici Edinburgensis Cognitarum CIII–CL. Notes from the Royal Botanic Garden Edinburgh 8(40): 313–348.

Song X, Song B, Fu M, Wang J, Liu J, Qin W, Jiang Y, Fan L, Yang B (2024) *Impatiens yingjingensis* (Balsaminaceae), a new species from Sichuan, China. PhytoKeys 242: 293–306. https://doi.org/10.3897/phytokeys.242.119702

Suksathan P, Triboun P (2009) Ten new species of *Impatiens* (Balsaminaceae) from Thailand. Gardens’ Bulletin Singapore 61(1): 159–184.

Tardieu-Blot ML (1944) Les *Impatiens* d’indochine, repartition, affinites et description d’especes nouvelles. Notulae Systematicae (Paris) 11(4): 169–185.

Tharani R, Murugesan M, Ravichandran V, Karthik B, Anusuba V (2021). Rediscovery of *Impatiens laticornis* CEC Fisch. (Balsaminaceae), a stenoendemic and critically endangered species from Nilgiri Biosphere Reserve, southern India. Nelumbo 63(2): 32–36. <https://doi.org/10.20324/nelumbo/v63/2021/167714>

Thwaites GHK (1864) Enumeratio plantarum Zeylaniae: an enumeration of Ceylon plants, with descriptions of the new and little-known genera and species, observation on their habitats, uses, native names, etc. John Edward printers, London, 483 pp.

Toppin SM (1920) Notes on the Balsams of Chitral and the Kachin Hills. Bulletin of Miscellaneous information, Kew 10: 345–367.

Utami N, Shimizu T (2005) Seed morphology and classification of *Impatiens* (Balsaminaceae). Blumea 50(3): 447–456. https://doi.org/10.3767/000651905X622699

Warburg O 1897 Balsaminaceae Africanae Botanische Jahrbücher für Systematik, Pflanzengeschichte und Pflanzengeographie 22(2): 46–53.

Wight R (1837) On the genus *Impatiens*. Madras Journal of Literature and Science 5(15): 1–15.

Wight R, Walker-Arnott GA (1834) Prodromus Flora Peninsula Kindle Orientalis: Containing Abridged Descriptions Of The Plants Found In The Peninsula Of British India, Arranged According To The Natural System Vol. I. London Parbury, Allen, 1−480.

Xu YL, Chen YL (1999) New taxa of *Impatiens* L. (Balsaminaceae) from Zhejiang, China. Journal of Systematics and Evolution 37(2): 194–200.

Yuan YM, Song Y, Ge XJ (2011) *Impatiens qingchengshanica* (Balsaminaceae), a unique new species from China and its phylogenetic position. Botanical Studies 52(2): 225–230.

Zeng L, Liu YN, Gogoi R, Zhang LJ Yu SX (2015) *Impatiens tianlinensis* (Balsaminaceae), a new species from Guangxi, China. Phytotaxa 227(3): 253–260. <https://doi.org/10.11646/phytotaxa.227.3.4>

Zhu C-S, Wei H-W (1994) A new species of *Impatiens* (Balsaminaceae) from Henan. Bulletin of Botanical research 14(3): 243–245

Zika PF (2006) *Impatiens× pacifica* (Balsaminaceae), a new hybrid jewelweed from the Pacific Northwest coast of North America. Novon: A Journal for Botanical Nomenclature 16(3): 443–448. [https://doi.org/10.3417/1055-3177(2006)16[443:IPBANH]2.0.CO;2](https://doi.org/10.3417/1055-3177(2006)16%5b443:IPBANH%5d2.0.CO;2)
